# CXCL3 Antagonism Reverses Systemic Antitumor Immune Memory Impairment Associated With High Tumor Burden and Sensitizes Anti‐PD‐1 Therapy

**DOI:** 10.1002/advs.77976

**Published:** 2026-09-27

**Authors:** Ji Wang, Xinyu Tang, Zeju Li, Qi Qi, Chang Sun, Wen Sun, Weiya Zhang, Yue Yin, Ya Deng, Peiwen Ling, Yifan Xu, Guangshun Sun, Yi Ren, Hong Pan, Shui Wang, Wenbin Zhou

**Affiliations:** ^1^ Department of Breast Surgery The First Affiliated Hospital with Nanjing Medical University Nanjing China; ^2^ Department of Breast and Thyroid Surgery The Affiliated Huai'an No.1 People's Hospital of Nanjing Medical University Huai'an China; ^3^ Jiangsu Key Lab of Cancer Biomarkers Prevention and Treatment Jiangsu Collaborative Innovation Center for Cancer Personalized Medicine School of Public Health Nanjing Medical University Nanjing China

**Keywords:** CXCL3, immune checkpoint blockade, neutrophils, systemic antitumor immunity, T cells, tumor burden

## Abstract

High tumor burden is associated with poorer immunotherapy responses compared to low burden, yet the systemic mechanisms driving this impairment and strategies to reverse it remain unclear. We demonstrate that tumors with higher malignancy or stages exert a more pronounced suppression on systemic antitumor immunity. Critically, high tumor burden drives persistent defects in CD8+ T cell‐mediated immune memory even after tumor resection, compared to low burden. Through single‐cell RNA and T cell receptor sequencing in mouse models, we identify a subset of tumor‐reactive Cxcr6+Cd8+ T (TRT) cells whose early activation, expansion, and long‐term memory are suppressed in high burden settings, compared to those in low burden settings. Mechanistically, high‐burden tumors secrete more CXCL3 than low burden, which promotes expansion of TGFβ1+ neutrophils, thereby inhibiting the function of CD8+ TRT cells. Neutrophil depletion reverses this immunosuppression, and CXCL3 antagonism under adjuvant/neoadjuvant settings restores the activation and long‐term memory formation of CD8+ TRT cells impaired by high tumor burden. CXCL3 antagonism synergizes with anti‐PD‐1 therapy, enhancing tumor control in orthotopic and metastatic models. Collectively, the CXCL3‐neutrophil‐TGFβ1 axis is a key mediator of tumor burden‐induced systemic immune dysfunction, and CXCL3 antagonism represents a promising strategy to improve immunotherapy outcomes, particularly for patients with locally advanced or advanced disease.

## Graphical abstract

This study reveals that large tumors secrete more chemokine CXCL3, which recruits immunosuppressive neutrophils to the periphery, thereby impairing the activation and memory of systemic tumor‑reactive CD8+ T cells. CXCL3 antagonism restores systemic immune memory and enhances the antitumor efficacy of anti‑PD‑1 therapy, offering a potential strategy to improve immunotherapy outcomes in patients with high tumor burden. (Created with BioRender; https://BioRender.com/bkifkpm)

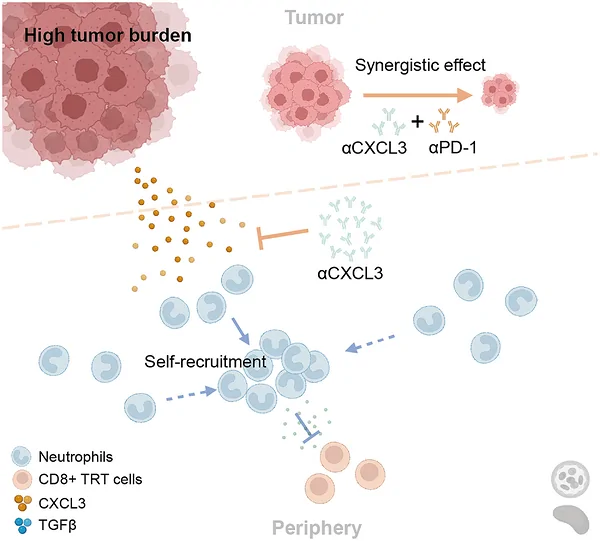

## 1 Introduction

Immune checkpoint blockade (ICB) therapy, particularly targeting PD‐1/PD‐L1, has been widely adopted in clinical oncology practice, yet clinical responses vary dramatically among patients [1, 2]. Patients with early‐stage tumors often exhibit favorable responses to neoadjuvant anti‐PD‐1/PD‐L1 therapy. In the CheckMate‐159 study, for untreated, resectable early‐stage non‐small‐cell lung cancer patients, neoadjuvant nivolumab (an anti‐PD‐1 monoclonal antibody) achieved a major pathological response rate of up to 45%, with treatment responses showing limited dependence on tumor PD‐L1 expression [3]. In the Neotorch study, toripalimab (an anti‐PD‐1 monoclonal antibody) or placebo was combined with platinum‐based chemotherapy, and the toripalimab group showed event‐free survival benefit regardless of PD‐L1 expression levels, including the < 1% subgroup, with a consistent efficacy trend [4]. However, patients with advanced‐stage cancer generally show poorer responses to ICB. In the KEYNOTE‐086 trial, the overall objective response rate to pembrolizumab (an anti‐PD‐1 monoclonal antibody) monotherapy was only 5.3% in patients with advanced triple‐negative breast cancer [5]. In the CheckMate‐057 study, nivolumab administered as second‐line therapy for advanced non‐squamous non‐small‐cell lung cancer yielded an objective response rate more than three times higher in PD‐L1‐positive patients than in PD‐L1‐negative patients [6]. The results from the PEARL and KEYNOTE‐024 studies further demonstrate the high dependence of ICB efficacy in advanced tumors on PD‐L1 expression [7, 8]. Therefore, compared to early‐stage tumors, the population benefiting from ICB therapy in advanced‐stage tumors is more limited.

Tumors are not just a local issue, and their occurrence and development disrupt the systemic immune landscape, forming a continuously communicating immune‐suppressive network [9, 10, 11]. Effective antitumor immunity relies not only on the local tumor microenvironment but also on the coordinated involvement of the systemic immune [12]. Accumulating evidence indicates that an intact systemic immune function is crucial for the success of ICB therapy [13, 14, 15]. Given the diminished efficacy of ICB in advanced settings, we hypothesize that high tumor burden may exert more pronounced effects on the remodeling of the tumor immune macroenvironment, thereby influencing treatment outcomes.

The activation of antitumor immunity and formation of immunological memory play a pivotal role in improving the prognosis of cancer patients, which can facilitate the elimination of recurrent and metastatic “seeds,” such as circulating tumor cells [16, 17, 18, 19, 20]. In previous clinical studies, the primary tumor burden remains an important prognostic factor, even after patients have undergone curative surgery [21, 22]. This suggests that the heterogeneity of systemic immune activation and memory induced by differential tumor burdens is persistent and plays a critical role in disease prognosis.

In this context, it is of significant value to comprehensively elucidate the dynamic impact of differential tumor burden on systemic immunity. Reshaping the systemic immune status under high tumor burden may represent an effective approach to enhance ICB efficacy. In this study, we revealed that CXCL3 derived from high tumor burden mediates expansion of systemic immunosuppressive TGFβ+ neutrophils, which impairs the activation of antitumor CD8+ T cells and consequently compromises the long‐term memory phenotype formation; CXCL3 antagonism reverses this effect and synergizes with anti‐PD‐1 therapy. While breast cancer serves as the primary model system in this study, the inclusion of the MC38 colon adenocarcinoma model allowed us to test the generalizability of our findings across distinct tumor types.

## 2 Results

### 2.1 Highly Malignant Tumors Disrupt Systemic CD8+ T Cell Immunity

**FIGURE 1:**
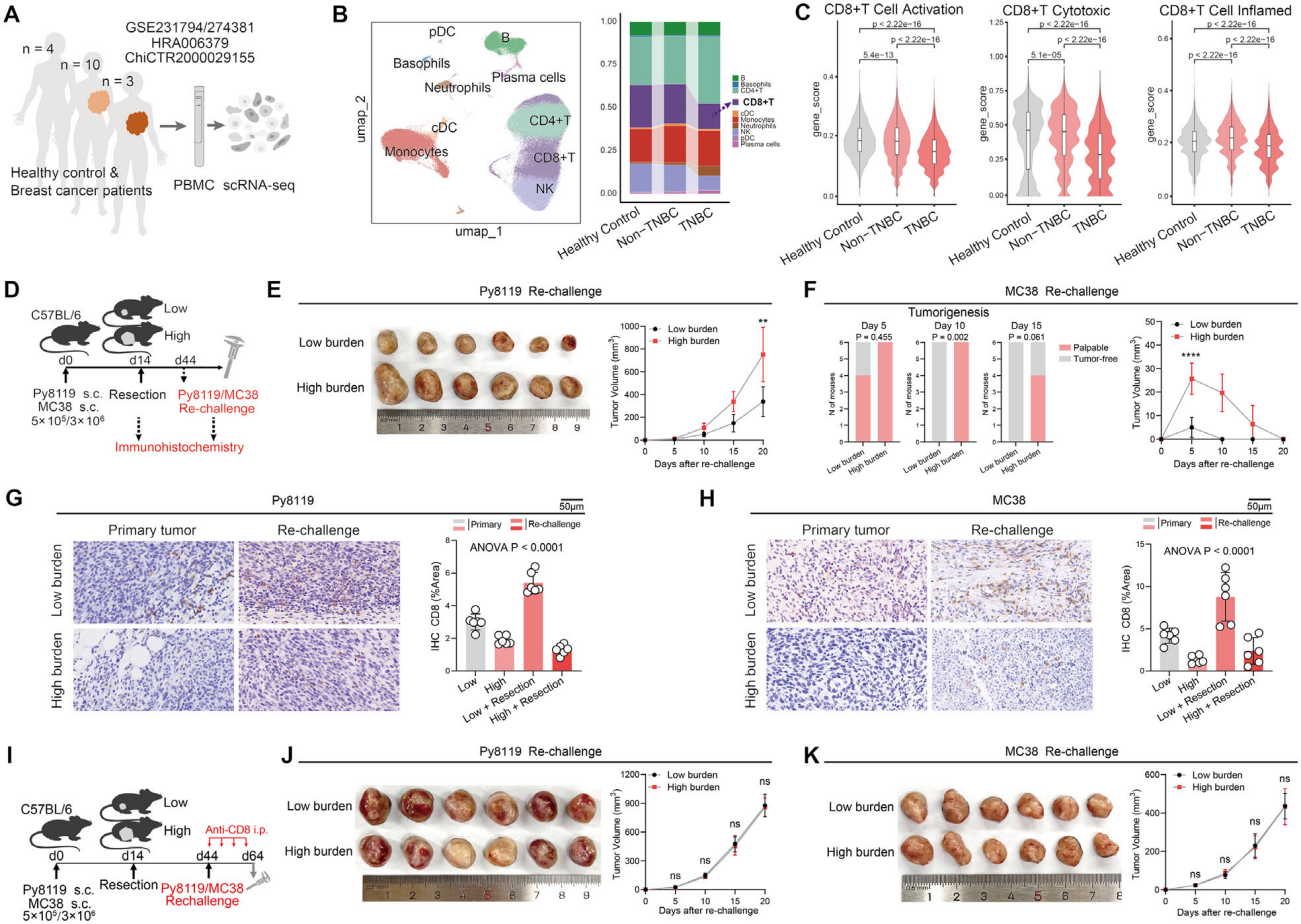
Impairment of systemic antitumor immune memory phenotypes in CD8+ T cells induced by high tumor burden. (A) Schematic diagram of data sources and sample sizes for single‐cell RNA sequencing of human peripheral blood mononuclear cells. (B) UMAP visualization of cell clusters and their proportions derived from single‐cell RNA sequencing data. (C) Comparison of gene set scores for CD8+ T cell activation, cytotoxicity, and inflammation among healthy controls, non‐TNBC, and TNBC groups in single‐cell RNA sequencing analysis. (D) Experimental design of tumor rechallenge after resection. (E) Tumor growth curves after rechallenge and tumor images on day 20 after rechallenge in Py8119 models following resection (*n* = 6 per group). (F) Tumor growth curves after rechallenge and tumorigenesis rates on day 5, 10, and 15 post‐rechallenge in MC38 models following resection (*n* = 6 per group). (G, H) Representative IHC images of CD8 expression in primary tumors and rechallenged tumors after resection, with comparative %Area analysis of CD8+ T cell infiltration in Py8119 and MC38 models (*n* = 6 per group). Scale bar: 50 µm. (I) Experimental design of tumor rechallenge after resection under CD8a depletion. (J, K) Tumor growth curves after rechallenge and tumor images on day 20 after rechallenge under CD8+ T cell exhaustion following resection in the Py8119 and MC38 models (*n* = 6 per group). ns not significant, ^*^ *p* < 0.05, ^**^ *p* < 0.01, ^***^ *p* < 0.001, ^****^ *p* < 0.0001. UMAP, uniform manifold approximation and projection; TNBC, triple‐negative breast cancer; IHC, immunohistochemistry.

We analyzed single‐cell RNA sequencing (scRNA‐seq) data of peripheral blood immune cells from non‐metastatic breast cancer patients and healthy controls across multiple clinical studies (Figure 1A) [23, 24, 25, 26]. Based on molecular subtypes, breast cancer patients were categorized into triple‐negative breast cancer (TNBC) and non‐triple‐negative breast cancer (non‐TNBC) groups, as these two subtypes exhibit obvious differences in malignancy. The distribution of tumor stages was comparable between these molecular subtypes. The results revealed that TNBC patients showed a significantly lower proportion of CD8+ T cells in peripheral blood compared to healthy controls and non‐TNBC patients (Figure 1B). Furthermore, compared to healthy controls, breast cancer patients exhibited significantly reduced activation and cytotoxicity scores in CD8+ T cells, with TNBC patients showing more pronounced reductions (Figure 1C), potentially related to the highly malignant nature of TNBC. The T cell inflamed score in TNBC patients was also significantly lower than the other two groups (Figure 1C). Notably, compared to healthy controls, both exhaustion and memory scores of CD8+ T cells from non‐TNBC patients were reduced (Figure S1A). These findings confirmed that the presence of tumors disrupts systemic immunity, particularly in highly aggressive tumors.

### 2.2 High Tumor Burden Impairs Systemic Immune Memory

To evaluate the impact of differential tumor burden on systemic long‐term antitumor immune memory, we compared CD8+ T cell function between pT1 and pT2 patients, and found that, compared with pT1 patients, pT2 patients exhibited higher exhaustion scores and lower activation and progenitor exhaustion scores (Figure S1B). These exploratory human observations prompted the hypothesis that tumor burden, in addition to subtype‐specific biology, may contribute to systemic CD8+ T cell dysfunction. Thus, we utilized Py8119 and MC38 cell lines, which represent high malignancy, to establish mouse models with low tumor burden (low burden) and high tumor burden (high burden), respectively. Following resection of primary tumors with low or high burdens, mice were rechallenged with the same tumor cells on the contralateral side (Figure 1D). In the high burden group, mice exhibited significantly more accelerated tumor growth rates upon tumor rechallenge, consistent across both models (Figure 1E,F). In the Py8119 model, the tumor size on day 20 after rechallenge was significantly larger in the high burden group compared to the low burden group (Figure 1E). In the MC38 model, both groups eventually achieved complete regression of rechallenged tumors (Figure 1F). However, the high burden group exhibited a significantly larger maximum tumor volume compared to the low burden group, along with a higher proportion of palpable tumors on days 5, 10, and 15 after rechallenge (Figure 1F). Additionally, the time from peak volume to complete regression was longer in the high burden group (Figure 1F). Immunohistochemical analysis was performed on primary tumors and rechallenged tumors at day 8 in both Py8119 and MC38 models. The results demonstrated that tumors in the high burden group exhibited a significantly lower proportion of CD8+ T cells compared with the low burden group at baseline (Figure 1G,H). Notably, rechallenged tumors in the high burden group also demonstrated a markedly reduced CD8+ T cell infiltration relative to those in the low burden group (Figure 1G,H). Moreover, in the low‐burden group, resection of the primary tumor led to increased CD8+ T cell infiltration in rechallenged tumors compared to primary tumors. This increase was absent in the high burden group (Py8119 model) or only marginal (MC38 model) (Figure 1G,H). Following in vivo depletion of CD8+ T cells (Figure 1I and Figure S1C), no significant differences were observed in the growth rate or volume of rechallenged tumors between the high and low burden groups in either the Py8119 or MC38 models (Figure 1J,K), indicating the pivotal role of CD8+ T cells in driving the divergent immune memory phenotypes between these two groups. In the Py8119 model, following tumor resection and subsequent tail vein injection of syngeneic tumor cells, the high burden group developed significantly more pulmonary metastases than the low burden group (Figure S1D,E). To exclude confounding factors, we additionally established low and high burden models by inoculating the same number of Py8119 cells and allowing tumors to grow until day 14 (low burden) and day 28 (high burden), respectively. The observed differences in tumor rechallenge were consistent with those obtained using the previous modeling approach (Figure S1F). Therefore, compared to those with low burden, tumors with high burden fail to establish robust CD8+ T cell‐mediated systemic long‐term immune memory.

### 2.3 Cxcr6+Cd8+ TRT Cells Represent Key Tumor‐Reactive T Cells

To exclude the potential influence of the tumor resection procedure itself on the differential systemic antitumor immune memory between the low and high burden groups, we analyzed the physiological status, inflammatory responses, and liver function of mice at day 3 post‐resection. Using Py8119 model as an example, we standardized the incision length to comparable levels between the low and high burden groups (Figure S2A). Additionally, the resected primary tumors from both Py8119 and MC38 models were presented, including specimens of tumors with low and high burden (Figure S2B). Additionally, no significant differences were observed between the two groups in body weight, C‐reactive protein levels, white blood cell counts, neutrophil percentage, lymphocyte percentage, monocyte percentage, alanine aminotransferase (ALT), or aspartate aminotransferase (AST) (Figure S2C). Thus, the tumor resection procedure per se is not the dominant factor underlying the differential long‐term systemic immune memory phenotypes observed between the low and high burden groups.

**FIGURE 2:**
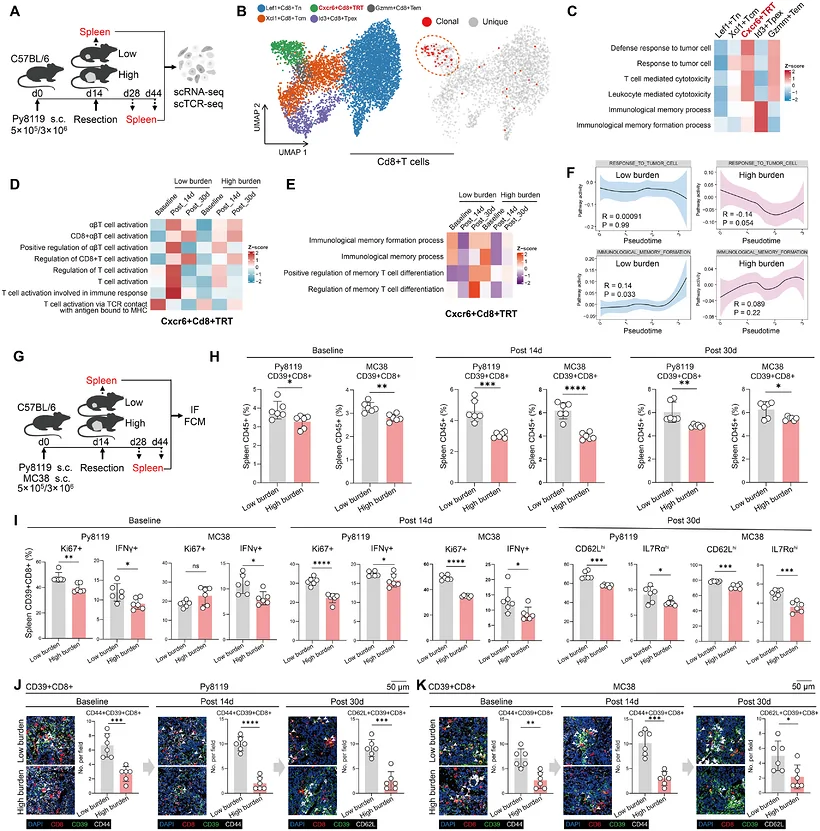
Impairment of early activation/proliferation and long‐term memory phenotypes in CXCR6+CD8+ tumor‐reactive T cells induced by high tumor burden. (A) Experimental design of single‐cell RNA and TCR sequencing in spleens of mice with low and high tumor burden at baseline, day 14 post‐resection, and day 30 post‐resection (*n* = 5 mice pooled per group). (B) UMAP visualization of Cd8+T cell subsets and their TCR clonalities (clonal or unique clones) in single‐cell sequencing analysis. (C) Heatmap showing scores of tumor response, cytotoxicity, and immune memory across Cd8+T cell subsets in single‐cell sequencing analysis. (D) Comparison of T‐cell activation pathway functional scores in Cxcr6+Cd8+TRT cells in the low and high burden groups at the three time points in A). (E) Comparison of T‐cell memory pathway functional scores in Cxcr6+Cd8+TRT cells in the low and high burden groups at the three time points in A). (F) Trends of tumor response and memory formation scores of Cxcr6+Cd8+TRT cells along pseudotime trajectories after resection in the low and high burden groups. (G) Experimental design of flow cytometry analysis for splenic immune cells in the low and high burden tumor‐bearing mice at baseline, day 14 post‐resection, and day 30 post‐resection. (H) Flow cytometry analysis comparing the percentages of splenic CD39+CD8+T cells among CD45+ cells between the low and high burden groups at three time points in G) (*n* = 6 per group). (I) Flow cytometry analysis comparing the percentages of Ki67+ and IFNγ+ cells among splenic CD39+CD8+ T cells between the low and high burden groups at baseline and day 14 post‐resection, as well as the percentages of CD62L^hi^ and IL7Rα^hi^ cells at day 30 post‐resection (*n* = 6 per group). (J, K) Immunofluorescence staining images of CD44+CD39+CD8+ T cells at baseline and day 14 post‐resection, and CD62L+CD39+CD8+ T cells at day 30 post‐resection in the spleens of mice with high vs. low tumor burden in Py8119 and MC38 models (*n* = 6 per group). The bar graphs show a comparison of the numbers of positive cells per field of view between the two groups at three time points. White arrows indicate the aforementioned cells. Scale bar: 50 µm. ns not significant, ^*^ *p* < 0.05, ^**^ *p* < 0.01, ^***^ *p* < 0.001, ^****^ *p* < 0.0001. TCR, T cell receptor; UMAP, uniform manifold approximation and projection; TRT cells, tumor‐reactive T cells.

The above results demonstrate that systemic antitumor immune alterations induced by tumors with different burdens are persistent and distinct, even after complete tumor resection. These finding also underscores the importance of dynamic and holistic analysis of systemic antitumor immunity both at baseline and following tumor resection. Mouse spleens were harvested for analysis, as the immune cell status in the spleen serves as a reliable indicator of systemic immunity [27]. We performed scRNA‐seq and single‐cell T cell receptor sequencing (scTCR‐seq) on spleen immune cells from the low and high burden groups at baseline, day 14 post‐resection, and day 30 post‐resection (Figure 2A). After standard data processing and quality control procedures, 56 203 scRNA‐seq profiles were classified, including 30 521 B cells, 9658 Cd4+ T cells, 6033 myeloid cells, 6779 Cd8+ T cells, 1752 natural killer (NK) cells, 1014 γδT cells, and 446 plasma cells (Figure S3A,B). The characteristic marker genes of these cell clusters were shown in Figure S3C.

First, the activation and memory phenotypes of overall Cd8+ T cells from the low and high burden groups were analyzed at baseline, day 14 post‐resection, and day 30 post‐resection. The results revealed no obvious differences in functional scores related to immune activation of overall Cd8+ T cells between the two groups at three time points (Figure S3D). The scores related to immune memory of overall Cd8+ T cells in the high burden group were slightly lower than those in the low burden group at day 30 post‐resection, but no notable differences were observed at baseline or day 14 post‐resection (Figure S3E). Hence, the functional profiles of overall CD8+ T cells only weakly reflect the disparities in systemic antitumor immune phenotypes between the low and high burden groups, necessitating further subpopulation analysis of CD8+ T cells.

Subsequent subclustering of Cd8+ T cells identified five distinct subsets: Lef1+Cd8+ T cells, Cxcr6+Cd8+ T cells, Gzmm+Cd8+ T cells, Xcl1+Cd8+ T cells, and Id3+Cd8+ T cells (Figure 2B). Among these, Cxcr6+Cd8+ T cells accounted for nearly all expanded TCR clonotypes (Figure 2B) and were therefore defined as tumor‐reactive T (TRT) cells. Based on the characteristic marker gene expression profiles of these Cd8+ T cell subsets, they were reclassified and renamed as follows: Lef1+Cd8+ Tn cells (naïve), Cxcr6+Cd8+ TRT cells (tumor‐reactive), Gzmm+Cd8+ Tem cells (effector memory), Xcl1+Cd8+ Tcm cells (central memory), and Id3+Cd8+ Tpex cells (precursor exhausted) (Figure 2B and Figure S3F). Compared to other subsets, Cxcr6+Cd8+ TRT cells characteristically expressed Cxcr6 and Entpd1 (CD39), exhibited the highest tumor reactivity and cytotoxicity, and showed immune memory scores comparable to or slightly higher than those of Xcl1+Cd8+ Tcm cells (Figure 2C and Figure S3F). Pathway enrichment analysis revealed that Cxcr6+Cd8+ TRT cells functionally resemble tissue‐resident memory T cells (Figure S3G) [28]. Collectively, Cxcr6+Cd8+ TRT cells represent functionally defined Cd8+ T cells with a systemic antitumor immune phenotype.

### 2.4 High Tumor Burden Restricts Systemic CD8+ TRT Cell Activation and Memory Formation

Given the critical role of Cxcr6+Cd8+ TRT cells in systemic antitumor immunity, we focused on analyzing the functional differences of this subset between the low and high burden groups. At baseline, the activation‐related scores of Cxcr6+Cd8+ TRT cells in the high burden group were slightly reduced compared to those in the low burden group (Figure 2D). By day 14 post‐resection, representing the early phase after resection, the activation‐related scores of these cells were evidently lower in the high burden group relative to those in the low burden group (Figure 2D). At day 30 post‐resection, corresponding to the long‐term phase, the scores associated with immunological memory formation were markedly reduced in the high burden group compared to those in the low burden group (Figure 2E). At all three time points, the high burden group exhibited slightly lower distribution proportions of high scores for tumor reactivity, cytotoxicity, and clonal expansion in Cxcr6+Cd8+ TRT cells compared to the low burden group (Figure S3H). Cxcr6+Cd8+ TRT cells from low and high burden groups showed comparable expression of exhaustion markers (Pdcd1, Havcr2, Lag3) at three time points, indicating that T cell exhaustion is unlikely to be the dominant driver of the distinct early activation and memory phenotypes (Figure S3I).

Pseudotime analysis revealed a slightly reduced differentiation tendency of Cd8+ T cells toward Cxcr6+Cd8+ TRT cells in the high burden group compared to the low burden group during the early post‐resection phase (Figure S3J). Additionally, both at baseline and in the early phase after resection, the proportion of clonally expanded Cxcr6+Cd8+ TRT cells was significantly lower in the high burden group than that in the low burden group (Figure S3J). The dynamic changes in functional scores of Cxcr6+Cd8+ TRT cells were analyzed separately following tumor resection. The results showed that the tumor reactivity scores of Cxcr6+Cd8+ TRT cells in the high burden group decreased along the pseudotime trajectory (*p* = 0.054), while those in the low burden group remained relatively stable (*p* = 0.99) (Figure 2F). The immunological memory formation scores of these cells in the high burden group showed minimal change over pseudotime (*p* = 0.22), whereas scores in the low burden group increased progressively, particularly during the long‐term phase (*p* = 0.033) (Figure 2F). Both groups exhibited an increasing trend in cytotoxicity scores (high burden: *p* = 0.0041; low burden: *p* = 0.00013), although the increase was more attenuated in the high burden group compared to that in the low burden group (Figure S3K).

To validate the findings from the scRNA/TCR‐seq, we harvested spleens from Py8119 and MC38 tumor‑bearing mice at baseline, day 14 post‑resection, and day 30 post‑resection for flow cytometry (Figure 2G). In the scRNA/TCR‐seq results, Cxcr6+Cd8+ TRT cells with antitumor effects were characterized by high expression of Cxcr6 and Entpd1 (CD39) (Figure S3F). Given that CD39 protein expression is relatively more stable and more dependent on antigen‐specific TCR engagement [29], it was selected as the tumor‐reactive marker in flow cytometry.

In Py8119 model, at baseline, the proportion of overall CD8+ T cells and CD39+CD8+ T cells was significantly lower in the high burden group than in the low burden group (Figure 2H and Figure S4A). Additionally, the expression of activation markers, IFNγ and Ki67, in CD39+CD8+ T cells was significantly reduced in the high burden group compared to the low burden group (Figure 2I). These differences persisted at day 14 post‐resection (Figure 2I). Consistent results were observed in the MC38 model, except that no significant difference in Ki67 expression in CD39+CD8+ T cells was detected between the two groups at baseline (Figure 2H,I and Figure S4A). Collectively, high tumor burden results in insufficient early activation and proliferation of systemic CD39+CD8+ T cells with tumor reactivity in the early phase.

At day 30 post‐resection, no significant differences were observed in the proportions of overall CD8+ T cells between the two groups in either Py8119 or MC38 models (Figure S4A). However, the proportion of CD39+CD8+ T cells was significantly lower in the high burden group, accompanied by reduced expression of memory markers, CD62L and IL7Rα, in these cells compared to the low burden group (Figure 2H,I). Furthermore, the frequency of CD44^hi^CD62L^hi^CD8+ T cells with a central memory phenotype within the CD39+CD8+ T cell population was significantly decreased in the high burden group relative to the low burden group (Figure S4B,C). These results indicate that, even after complete tumor resection, high tumor burden can lead to impaired systemic long‐term antitumor immune memory.

To intuitively demonstrate the dynamic changes in systemic antitumor immunity under low and high tumor burden at three time points, we employed multiplex immunofluorescence (mIF) staining to compare the functional phenotypes of splenic CD8+ TRT cells in two models. At baseline and day 14 post‐resection, the proportion of CD44+CD39+CD8+ T cells with an activated phenotype was consistently lower in the high burden group compared to the low burden group (Figure 2J,K and Figure S4D,E). At day 14 post‐resection, the proportion of CD44+CD39+CD8+ T cells in the high burden group showed no obvious change compared to the baseline, with even a slight decrease (Figure 2J,K and Figure S4D,E). Correspondingly, at day 30 post‐resection, the high burden group exhibited a lower proportion of CD62L+CD39+CD8+ T cells with a long‐term memory phenotype compared to the low burden group (Figure 2J,K and Figure S4D,E). Consistent results were observed in both Py8119 and MC38 models. However, CD39+CD8+ T cells were scarcely detectable in primary tumors in both groups (Figure S4F). Notably, in rechallenged tumors, CD39+CD8+ T cells were abundant in the low burden group but remained extremely rare in the high burden group (Figure S4F), further supporting that CD39+CD8+ T cells primarily represent systemic antitumor immunity.

We noted that CD8 depletion alone establishes CD8 dependence, whereas the CXCR6 neutralization and adoptive transfer experiments specifically implicate the CXCR6+CD8+ TRT subset in the differential memory phenotypes. Therefore, to further define the role of Cxcr6+Cd8+ TRT cells in systemic antitumor immune memory, CXCR6 was neutralized during rechallenge in the low burden group, which abrogated protection and yielded tumor growth rates similar to those of the high burden group (Figure S4G). Adoptive transfer of splenic CXCR6+CD8+ TRT cells isolated from the low burden group into high burden mice rendered the rechallenged tumor growth in recipients comparable to that in the low burden group (Figure S4H). Adoptive transfer of splenic CXCR6+CD8+ TRT cells into naive recipients showed that TRT cells from high burden donors conferred significantly less protection against tumor challenge than those from low burden donors (Figure S4I). Therefore, the formation of a systemic long‐term antitumor immune memory phenotype is dependent on the early activation and expansion of Cxcr6+Cd8+ TRT cells (including both baseline and day 14 post‐resection), which represents a vulnerable node in high tumor burden settings, ultimately leading to impaired long‐term antitumor memory.

### 2.5 B and CD4+ T Cells Play a Minimal Role in the Differential Systemic Immune Phenotypes Across Tumor Burdens

To investigate the upstream mechanisms through which high tumor burden leads to impaired immune activation and memory phenotypes in systemic CD8+ TRT cells, we first analyzed the functions of B cells and CD4+ T cells between the two groups. It is worth noting that NK cells, γδT cells, and plasma cells accounted for minimal proportions in the scRNA/TCR‐seq data (Figure S3B), suggesting that these subsets have negligible effects on the functions of CD8+ TRT cells. Therefore, they were not subjected to further analysis.

Among overall B cells, Cd38+Cr2+ B cells were defined as tumor‐reactive B cells in the scRNA/TCR‐seq [30, 31]. These tumor‐reactive B cells exhibited relatively higher expression of the memory marker Cd27 compared to non‐tumor‐reactive B cells. However, tumor‐reactive B cells accounted for a tiny proportion of overall B cells (Figure S5A–C). A comprehensive analysis of functional scores for overall B cells and tumor‐reactive B cells was conducted across the low and high burden groups. For overall B cells, no significant differences were observed between the two groups in activation, proliferation, or antigen processing and presentation scores, and so was the case for tumor‐reactive B cells (Figure S5D,E).

Following subclustering of overall Cd4+ T cells in the scRNA/TCR‐seq, five distinct subsets were identified: Lef1+Cd4+ T cells, Cxcr3+Cd4+ T cells, Maf+Cd4+ T cells, Foxp3+Cd4+ T cells, and Isg15+Cd4+ T cells (Figure S6A). Among these, expanded TCR clonotypes were predominantly distributed in Cxcr3+Cd4+ T cells, Maf+Cd4+ T cells, and Isg15+Cd4+ T cells (Figure S6A), indicating their association with tumor reactivity. Based on the characteristic marker gene expression profiles of these Cd4+ T cell subsets, they were reclassified and renamed as follows: Lef1+Cd4+ Tn cells (naïve), Cxcr3+Cd4+ Th1 cells (type 1 helper), Maf+Cd4+ Th2 cells (type 2 helper), Foxp3+Cd4+ Treg cells (regulatory), and Isg15+Cd4+ T cells (interferon‐stimulated) (Figure S6A,B). A comprehensive analysis of the proportions and functional scores of overall Cd4+ T cell subsets was performed in the low and high burden groups. The results revealed comparable distribution patterns of Cd4+ T cell subsets between the two groups (Figure S6C). No significant differences were observed in activation, proliferation, or cytokine production scores for overall Cd4+ T cells between the two groups, and so were the Cxcr3+Cd4+ Th1, Maf+Cd4+ Th2, and Isg15+Cd4+ T cells (Figure S6D–G).

These results exclude a significant contribution of B cells and CD4+ T cells to the disparate function of CD8+ TRT cells observed between the low and high burden groups.

### 2.6 High Tumor Burden Drives the Expansion of Systemic Immunosuppressive Neutrophils

**FIGURE 3:**
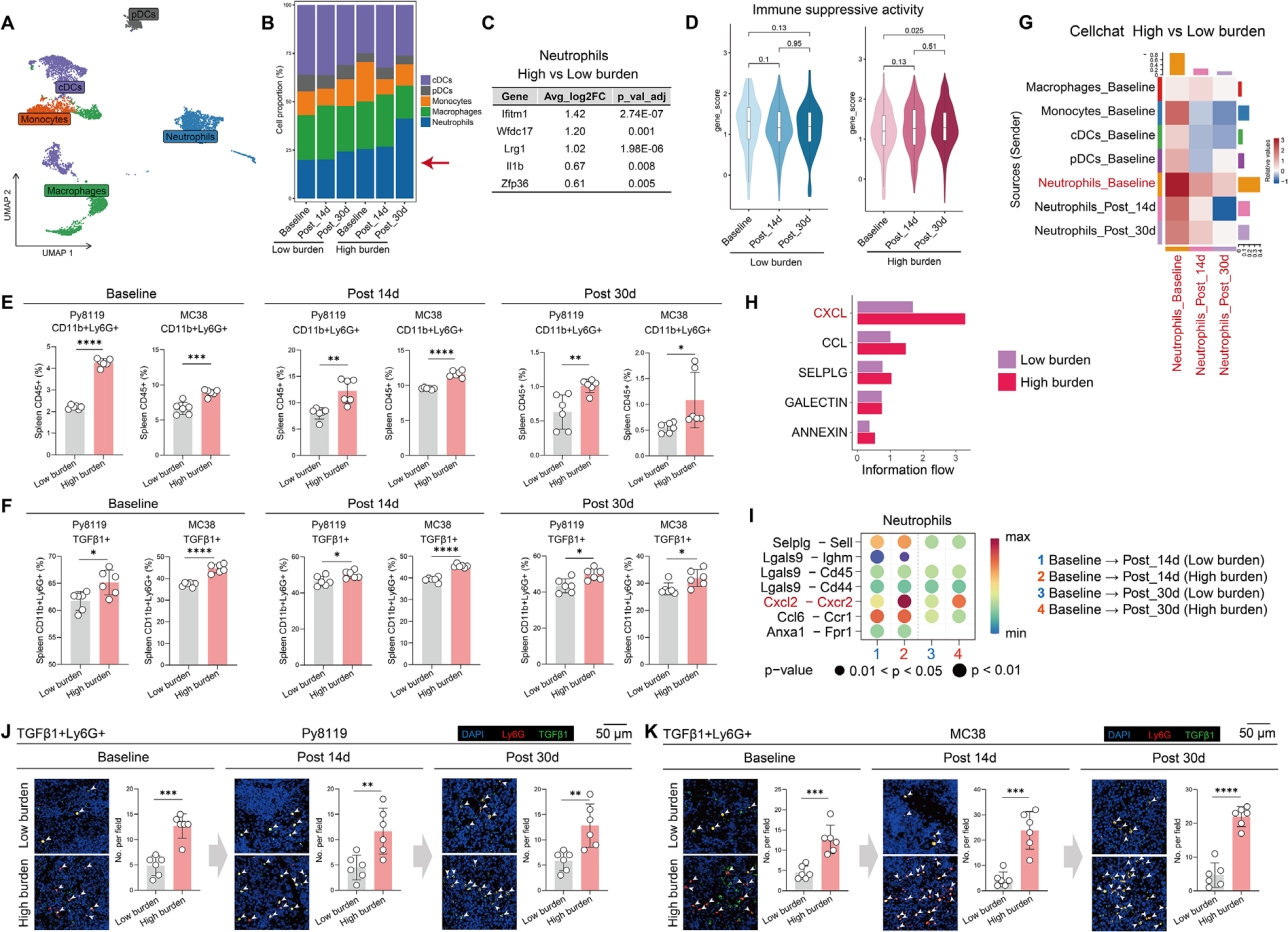
High tumor burden drives self‐recruitment and cascade amplification of systemic immunosuppressive neutrophils. (A) UMAP visualization of myeloid cell subsets in single‐cell sequencing analysis. (B) Percentages of different myeloid cell subsets in low vs. high burden groups at baseline, day 14 post‐resection, and day 30 post‐resection. (C) Comparison of immunosuppressive markers (Ifitm1, Wfdc17, Lrg1, Il1b, and Zfp36) in neutrophils between high and low burden groups at baseline. (D) Violin plots showing trends of neutrophil immunosuppressive activity scores in low and high burden groups across three time points in B). (E) Flow cytometry analysis comparing the percentage of splenic neutrophils among CD45+ cells between low and high burden groups at three time points in (B) (*n* = 6 per group). (F) Flow cytometry analysis comparing the percentage of splenic TGFβ1+ neutrophils among neutrophils between low and high burden groups at three time points in (B) (*n* = 6 per group). (G) Heatmap of CellChat interaction strength differences between high vs. low burden groups for crosstalk among myeloid subsets at baseline, neutrophils at day 14 post‐resection, and neutrophils at day 30 post‐resection. (H) Bar chart comparing information flow of neutrophils in low and high burden groups. (I) Dot plot comparing interaction strengths of ligand‐receptor pairs between neutrophils at baseline and day 14 or day 30 post‐resection in low and high burden groups. (J, K) Immunofluorescence staining images of immunosuppressive TGFβ1+Ly6G+ neutrophils in the spleen of mice with high vs. low tumor burden in Py8119 and MC38 models at baseline, day 14 post‐resection, and day 30 post‐resection (*n* = 6 per group). The bar graphs show a comparison of the number of TGFβ1+Ly6G+ neutrophils per field of view between the two groups at three time points. White arrows indicate the aforementioned cells. Scale bar: 50 µm. ns not significant, ^*^ *p* < 0.05, ^**^ *p* < 0.01, ^***^ *p* < 0.001, ^****^ *p* < 0.0001. For one‑way ANOVA, the Bonferroni post‑hoc test was applied. UMAP, uniform manifold approximation and projection.

Notably, at baseline, day 14 post‐resection, and day 30 post‐resection, the proportion of myeloid cells in the high burden group was higher than that in the low burden group according to scRNA/TCR‐seq data (Figure S3B), necessitating further analysis of myeloid cells. Based on characteristic gene expression profiles, myeloid cells were classified into the following subsets: neutrophils, macrophages, monocytes, conventional dendritic cells (cDCs), and plasmacytoid dendritic cells (pDCs) (Figure 3A and Figure S7A). First, at three time points, the proportion of neutrophils was elevated in the high burden group compared to the low burden group, and the high burden group exhibited a progressively increasing trend in neutrophil frequency with an accelerating rate, a phenomenon not observed in the low burden group (Figure 3B). At baseline, among all myeloid cell subsets, only neutrophils exhibited a significant difference in immune response negative regulation scores between the two groups (*p* = 0.032), with the high burden group showing significantly higher scores than the low burden group (Figure S7B). Therefore, the function of neutrophils was further analyzed across the three time points. At baseline, neutrophils in the high burden group exhibited significantly higher expression of immunosuppression‐related markers, including Ifitm1, Wfdc17, Lrg1, Il1b, and Zfp36, compared to those in the low burden group (Figure 3C). Moreover, at baseline, neutrophils in the high burden group exhibited stronger characteristics of myeloid‐derived suppressor cells (MDSCs) compared to those in the low burden group (Figure S7C). Dynamic analysis revealed that the immune suppressive activity scores of neutrophils in the high burden group progressively increased across the three time points (Post_30d vs. Baseline: *p* = 0.025), whereas no significant differences were observed in the low burden group (Figure 3D). Comparison of molecular markers potentially associated with suppression of CD8+ TRT cell function, including Cd274, S100a8, S100a9, Tgfb1, Arg1, and Il1b, between the low and high burden groups suggested that only Tgfb1 showed consistent differences between the two groups across all time points (Figure S7D). Flow cytometry using both Py8119 and MC38 models demonstrated that, at all three time points, the proportions of overall neutrophils and TGFβ1+ neutrophils in the spleen were significantly higher in the high burden group than the low burden group (Figure 3E,F), consistent with the scRNA/TCR‐seq data. Thus, the increase in immunosuppressive neutrophils and their release of TGFβ may play a pivotal role in the functional impairment of CD8+ TRT cells.

Subsequently, interactions between neutrophils and other myeloid cells, as well as inter‐neutrophil interactions, were analyzed. Compared to the low burden group, only neutrophils at baseline in the high burden group exhibited strong interactions with themselves and with neutrophils post‐resection, particularly during the early phase (day 14 post‐resection) (Figure 3G). This suggests that high tumor burden may induce enhanced self‐recruitment of neutrophils in the periphery, leading to the aforementioned cascade amplification of neutrophils. Information flow and ligand‐receptor pair analyses revealed that CXCL‐CXCR interactions dominated the crosstalk between neutrophils at baseline and day 14 post‐resection in the high burden group, with neutrophils at baseline showing a stronger propensity to interact with neutrophils post‐resection via CXCL2‐CXCR2 compared to the low burden group (Figure 3H,I and Figure S7E–G). Besides, neutrophils at baseline in the high burden group exhibited higher outgoing interaction strength compared to those in the low burden group (Figure S7H).

The mIF was employed to dynamically characterize changes in neutrophils in the Py8119 and MC38 models. At all three time points, the high burden group exhibited a higher proportion of TGFβ1+ neutrophils compared to the low burden group (Figure 3J,K and Figure S7I,J). The proportions of TGFβ1+ neutrophils at day 14 and day 30 post‐resection were obviously elevated relative to the baseline in the high burden group, indicating early initiation of self‐recruitment that led to long‐term accumulation of TGFβ1+ neutrophils. However, the low burden group showed only minimal changes in the proportion of TGFβ1+ neutrophils across the three time points (Figure 3J,K and Figure S7I,J). Consistent results were observed in both Py8119 and MC38 models.

Besides, the expression of immunosuppressive signatures (including Arg1, Cd274, Tgfb1, and Il1b) in other myeloid subsets remained largely comparable between the low and high burden groups at all time points (Figure S7K). In Py8119 tumors, macrophages (TGFβ1^lo^) predominated, while neutrophils were scarce and rarely contacted CD8+ T cells in both burden groups (Figure S7L). By contrast, splenic TGFβ1+ neutrophils were broadly adjacent to CD8+ T cells, especially in the high burden group, and constituted the primary source of TGFβ1 (Figure S7M). At baseline, the TGFβ1‐producing‐related pathway score of neutrophils was significantly higher than that of macrophages and Foxp3+Cd4+ Treg cells (Figure S7N). Thus, neutrophils, rather than macrophages, represent the key upstream modulator of systemic CD8+ T cell responses under different burdens. To elucidate the potential mechanism underlying TGFβ1‑mediated regulation of CD8+ TRT cells, we assessed Tcf7, Smad3, Eomes, and Tbx21 expression in CD8+ TRT cells. At baseline, CD8+ TRT cells showed higher levels of Tcf7, Smad3, and Eomes in the high burden group than those in the low burden group, with Tcf7 exhibiting the most pronounced upregulation (Figure S7O). Sorted CXCR6+CD8+ TRT cells treated with TGFβ1 in vitro exhibited significantly higher TCF7 expression compared to untreated controls (Figure S7O). In the primary tumor, neutrophils were sparse, and TGFβ1 expression by TGFβ1+ cells was comparable between the two groups; in the rechallenged tumor, neutrophils remained sparse, yet TGFβ1 expression was virtually absent in the low burden group but detectable in the high burden group (Figure S7P), indicating a persistently suppressive neutrophil phenotype under high burden. Analysis of the scRNA‑seq data from the clinical cohort (Figure 1A) revealed that pT2 patients had significantly lower CXCR6 and ENTPD1 (encoding CD39) expression in CD8+ T cells, and higher TGFB1 expression in neutrophils, compared with pT1 patients (Figure S7Q), consistent with the findings from the mouse scRNA‑seq data.

In summary, under high tumor burden, systemic neutrophils undergo self‐recruitment, leading to the cascade amplification of immunosuppressive TGFβ1+ neutrophils. This process impedes the early activation and proliferation of CD8+ TRT cells, ultimately resulting in the suppression of long‐term systemic memory phenotype formation.

### 2.7 Peripheral Blood Mirrors Systemic Immune Impairment by High Tumor Burden

Similar to the spleen, immune cells in peripheral blood also serve as effective indicators of systemic immune [32, 33]. Peripheral blood from mice was utilized to validate the phenotypes of CD8+ T cells and neutrophils (Figure S8A). At baseline, compared to the low burden groups, the high burden groups exhibited reduced proportions of overall CD8+ T cells and CD39+CD8+ T cells in peripheral blood, along with decreased Ki67 expression in both Py8119 and MC38 models (Figure S8B–D). At day 14 post‐resection, significant differences in the proportions of overall CD8+ T cells and CD39+CD8+ T cells, as well as in the activation of CD39+CD8+ T cells, persisted in peripheral blood between the two groups (Figure S8B–D). At day 30 post‐resection, although the proportions of overall CD8+ T cells returned to comparable levels between the two groups, and the proportions of CD39+CD8+ T cells also showed no significant difference in the Py8119 model, the expression of memory markers CD62L and IL7Rα in CD39+CD8+ T cells in peripheral blood was significantly lower in the high burden group compared to the low burden group (Figure S8B–D). The proportion of CD44^hi^CD62L^hi^CD8+ cells among CD39+CD8+ T cells in peripheral blood was significantly lower in the high burden group (Figure S8E). Furthermore, the proportions of both overall neutrophils and TGFβ1+ neutrophils in peripheral blood were significantly higher in the high burden group compared to the low burden group across all three time points (Figure S8F–G). Collectively, findings from both the spleen and peripheral blood demonstrate that high tumor burden causes impairment of systemic antitumor immunity, characterized by suppressed early‐phase activation leading to compromised long‐term memory formation.

### 2.8 Neutrophil Depletion Reverses Immune Impairment of CD8+ TRT Cells

**FIGURE 4:**
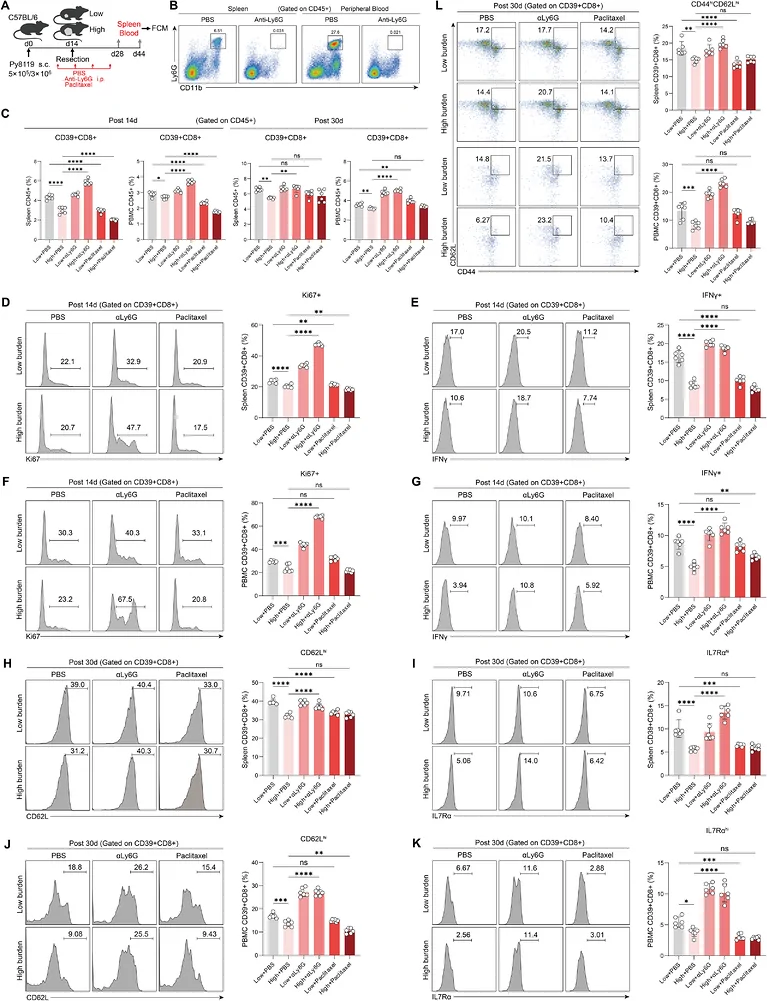
Effects of neutrophil depletion and paclitaxel treatment on early activation and long‐term memory phenotype of tumor‐reactive CD8+T cells. (A) Experimental design of neutrophil depletion and paclitaxel treatment. (B) Flow cytometry validation of neutrophil depletion efficacy in spleen and peripheral blood. (C) Comparison of CD39+CD8+ T cell percentages among CD45+ cells in spleen and peripheral blood across treatment groups at day 14 and day 30 post‐resection (*n* = 6 per group). (D–G) Comparison of Ki67+ and IFNγ+ cell percentages among CD39+CD8+T cells in spleen and peripheral blood across treatment groups at day 14 post‐resection (*n* = 6 per group), with representative histograms. (H–K) Comparison of CD62L^hi^ and IL7Rα^hi^ cell percentages among CD39+CD8+T cells in spleen and peripheral blood across treatment groups at day 30 post‐resection (*n* = 6 per group), with representative histograms. (L) Comparison of CD44^hi^CD62L^hi^ cell percentages among CD39+CD8+T cells in spleen and peripheral blood across treatment groups at day 30 post‐resection (*n* = 6 per group), with representative pseudocolor plots. ns not significant, ^*^ *p* < 0.05, ^**^ *p* < 0.01, ^***^ *p* < 0.001, ^****^ *p* < 0.0001. For one‑way ANOVA, the Bonferroni post‑hoc test was applied.

Given the critical role of neutrophils in shaping phenotypes of systemic CD8+ TRT cells, we attempted to deplete or reduce the proportion of neutrophils in mice, aiming to reverse the phenotypic alterations. Accordingly, we selected systemic administration of the anti‐Ly6G neutralizing antibody or paclitaxel around the time of tumor resection (Figure 4A,B), the latter of which can induce neutropenia through myelosuppression [34]. Following neutrophil depletion, the proportion of CD39+CD8+ T cells in the high burden group was significantly increased compared to the PBS group at both day 14 and day 30 post‐resection (Figure 4C). However, paclitaxel failed to significantly increase the proportion of CD39+CD8+ T cells in both the spleen and peripheral blood of the high burden group, and even resulted in a significant reduction compared to the PBS group at day 14 post‐resection (Figure 4C), which may be attributed to the additional suppressive effects of paclitaxel on lymphocyte activity [35, 36]. Moreover, neutrophil depletion significantly increased the proportions of Ki67+ and IFNγ+ cells within CD39+CD8+ T cells at day 14 post‐resection in the high burden group (Figure 4D–G). In contrast, paclitaxel not only failed to produce this effect but even suppressed the expression of activation markers in CD39+CD8+ T cells (Figure 4D–G). Consequently, neutrophil depletion markedly enhanced the proportions of CD62L^hi^, IL7Rα^hi^, and CD44^hi^CD62L^hi^CD8+ cells among CD39+CD8+ T cells at day 30 post‐resection in the high burden group (Figure 4H–L). Paclitaxel again showed no such effect and, notably, resulted in downregulation of these memory markers in the low burden group (Figure 4H–L).

We further compared the effects of neutrophil vs. macrophage depletion in the high burden model, and found that neutrophil depletion, but not macrophage depletion, rescued CD8+ TRT cell activation and memory (Figure S7R,S). Besides, expression of antigen presentation, costimulation, and maturation markers was largely comparable across all groups in both DC subsets (Figure S7T). Therefore, merely reducing neutrophil proportions through chemotherapy such as paclitaxel in the early phase fails to effectively enhance the long‐term memory phenotype of systemic CD8+ TRT cells, which may be related to the detrimental effects of chemotherapy on lymphocyte function. Nevertheless, selective neutrophil depletion alone significantly ameliorates the systemic long‐term antitumor immune memory phenotype impaired by high tumor burden.

### 2.9 CXCL3 Derived From High Tumor Burden Triggers Expansion of Peripheral Immunosuppressive Neutrophils

**FIGURE 5:**
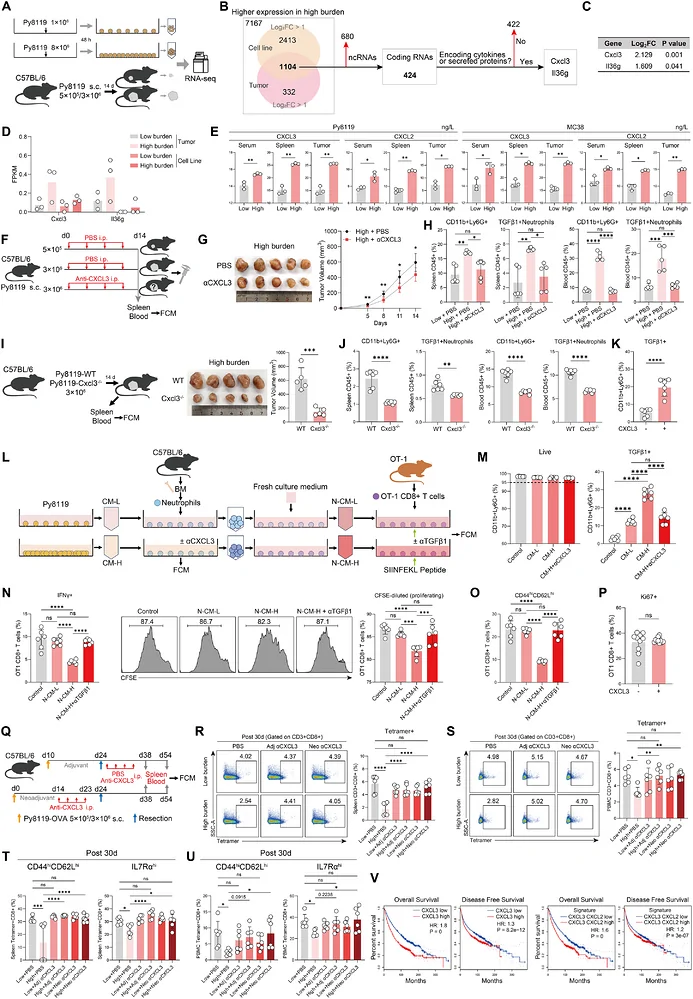
Restorative effect of CXCL3 antagonism on impaired systemic tumor‐specific CD8+ T cell functional phenotypes induced by high tumor burden. (A) Experimental design of bulk RNA sequencing for in vitro (cell line) simulated and in vivo (tumor) high vs. low burden tumors (*n* = 3 per group). (B) In bulk RNA sequencing, genes with log2FC > 1 in both in vitro and in vivo high burden groups (vs. low burden groups) were intersected, followed by exclusion of non‐coding RNAs to screen for potential cytokine‐ or secreted protein‐encoding genes. (C) The log2FC values and corresponding *p*‐values of Cxcl3 and Il36g expression in high vs. low burden groups in tumor tissues from in vivo models. (D) FPKM values of Cxcl3 and Il36g in low and high burden groups under in vivo and in vitro conditions. (E) Bar graphs comparing concentrations of CXCL2 and CXCL3 in serum, spleen, and tumor tissues between low and high burden groups by ELISA (*n* = 3 per group). (F) Experimental design of CXCL3 antagonism in controlling high burden tumors and remodeling systemic neutrophil phenotypes using the Py8119 model (*n* = 5 per group). (G) Tumor growth curves and tumor images following inoculation with 3 × 10^6^ Py8119 cells and treatment with PBS or αCXCL3 (*n* = 5 per group) in F). (H) Bar graphs comparing the proportions of neutrophils and TGFβ1+ neutrophils in the spleens and peripheral blood among the Low burden + PBS, High burden + PBS, and High burden + αCXCL3 groups (*n* = 5 per group) in F). (I) Experimental design of Py8119‑Cxcl3^−/−^ vs. wild‑type high burden subcutaneous tumor models, and tumor volumes and images of Py8119‑Cxcl3^−/−^ vs. wild‑type tumors (*n* = 5 per group). (J) Bar graphs comparing the proportions of neutrophils and TGFβ1+ neutrophils in the spleens and peripheral blood between Py8119‑Cxcl3^−/−^ and wild‑type groups (*n* = 6 per group). (K) Comparison of TGFβ1+ neutrophil proportions following in vitro treatment with or without recombinant CXCL3. (L) Experimental design of in vitro conditioned medium treatment of neutrophils and CD8+ T cells (*n* = 6 per group). (M) Comparison of neutrophil viability and TGFβ1+ neutrophil proportions following treatment with conditioned media from low and high burden Py8119 cultures (CM‑L and CM‑H), with or without αCXCL3. (N) Treatment of SIINFEKL‑stimulated OT1 CD8+ T cells with conditioned media from neutrophils cultured under low or high burden conditions (N‑CM‑L or N‑CM‑H). Comparison of IFNγ+ cells and CFSE dilution profiles among OT1 CD8+ T cells after treatment with N‑CM‑L or N‑CM‑H, with or without TGFβ1 neutralization (*n* = 6 per group). (O) Comparison of CD44^hi^CD62L^hi^ cells among OT1 CD8+ T cells in N) (*n* = 6 per group). (P) Comparison of Ki67+ cell frequencies among SIINFEKL‑stimulated OT1 CD8+ T cells treated with or without recombinant CXCL3 (*n* = 10 per group). (Q) Experimental design for analyzing systemic tumor‐specific CD8+ T cell immune activation and memory phenotypes via flow cytometry following CXCL3 antagonism in low and high burden groups (Py8119‐OVA model) under adjuvant and neoadjuvant settings. (R, S) Bar graph and representative pseudocolor plots comparing the proportions of tetramer+CD8+ T cells in spleens and peripheral blood across different groups at day 30 post‐resection, following systemic administration of PBS or CXCL3 antagonism under adjuvant/neoadjuvant settings (*n* = 6 per group). (T, U) Bar graph comparing the proportions of CD44^hi^CD62L^hi^ cells and IL7Rα^hi^ cells among OVA‐specific (tetramer+) CD8+ T cells in spleens and peripheral blood across different groups at day 30 post‐resection, following systemic administration of PBS or CXCL3 antagonism under adjuvant/neoadjuvant settings (*n* = 6 per group). (V) Associations of CXCL3 expression and CXCL2/CXCL3 gene signature with overall survival and disease‐free survival across all cancer types in the TCGA database. ns not significant, ^*^ *p* < 0.05, ^**^ *p* < 0.01, ^***^ *p* < 0.001, ^****^ *p* < 0.0001. For one‑way ANOVA, the Bonferroni post‑hoc test was applied. FPKM, fragments per kilobase of transcript per million mapped reads; ELISA, enzyme‐linked immunosorbent assay; TCGA, The Cancer Genome Atlas Program.

Neutrophil depletion is clinically impractical for therapeutic applications. Therefore, it is particularly crucial to investigate why high tumor burden promotes self‐recruitment and immunosuppressive phenotypes of neutrophils in peripheral tissues. Low and high burden Py8119 mouse models were established, and tumor tissues were collected. Simultaneously, different counts of Py8119 cells were plated in culture dishes and incubated for 48 h to simulate low and high tumor burden in vitro, and cells were then harvested. Both the in vitro simulated tumors and the in vivo generated tumors were subjected to RNA sequencing (Figure 5A). In both in vivo and in vitro settings, there were 7167 overlapping differentially expressed genes upregulated in the high burden group compared to the low burden group. From these, genes with Log_2_FC > 1 in either in vitro cells or in vivo tumor tissues were extracted, and their intersection was taken, resulting in 1104 shared genes. After removing non‐coding RNAs and filtering for genes potentially encoding cytokines or secreted proteins, two potential key differentially expressed genes were identified: Cxcl3 and Il36g (Figure 5B). Among these, Cxcl3 showed a Log2FC of 2.129 (*p* = 0.001), while Il36g exhibited a Log2FC of 1.609 (*p* = 0.041) in tumor tissues (Figure 5C). Moreover, both in vitro and in vivo, the average fragments per kilobase of transcript per million mapped reads (FPKM) values of Cxcl3 and Il36g were higher in the high burden group than in the low burden group. In vivo, the average FPKM value of Il36g was elevated in the high burden group compared to the low burden group; however, Il36g was barely expressed in either group under in vitro conditions (Figure 5D). Thus, it is unlikely that Il36g is expressed by tumor cells, but may originate from other immune or stromal cells. Instead, CXCL3 is likely the key cytokine released by high burden tumors that causes the impairment of systemic antitumor immune phenotypes. Previous studies have shown that, upon binding to CXCR2 of neutrophils, CXCL3 activates G protein‐coupled receptor signaling and upregulates the expression of chemokines such as CXCL2, and CXCL3 can reprogram neutrophils toward an N2‐like phenotype [37, 38]. At the protein level, in both Py8119 and MC38 models, the concentrations of CXCL3 and CXCL2 in serum, spleen, and tumor tissues were significantly higher in the high burden group compared to the low burden group (Figure 5E), validating the aforementioned findings. Compared with the low burden group, high burden mice exhibited significantly increased frequencies of both TGFβ1+CXCR2+ neutrophils and CD34+CD16/32+ granulocyte‑monocyte progenitors in the bone marrow, suggesting that the bone marrow serves as a potential source of the peripheral neutrophil expansion observed in the high burden setting (Figure S9A,B).

NF‑κB, JAK‑STAT, and HIF‑1 pathways were upregulated in high burden tumors in both in vitro and in vivo settings, consistent with their known regulation of CXCL3 [39, 40, 41]. The JAK‑STAT pathway showed significant activation in the in vitro high burden system (Figure S9C). At the protein level, high burden conditions increased p‑STAT3, total STAT3, and SOCS3 expression in both in vitro and in vivo models (Figure S9D), suggesting that JAK‑STAT3 activation may contribute to CXCL3 upregulation in high burden tumor cells. We further examined Cxcl9, Cxcl10, and Ccl5 to assess their potential contribution to CD8+ T cell recruitment and ICB response. Although these chemokines were upregulated in high burden tumor cells in vitro, no obvious differences were observed in vivo (Figure S9E), suggesting that their impact on systemic anti‑tumor immunity is limited and does not explain the burden‑dependent immune divergence. The splenic scRNA‑seq data revealed only minimal differences in the expression of Cxcl9, Cxcl10, and Ccl5 in myeloid cells, as well as Cxcr3 and Ccr5 on Cxcr6+Cd8+ TRT cells (Figure S9F,G), further supporting a dominant role for tumor‑derived CXCL3 in driving the systemic immune differences between high and low burden groups.

To elucidate the critical role of CXCL3 in peripheral tissues, tumor‐free mice were intravenously injected with recombinant CXCL3, using PBS as a control (Figure S9H). Compared to the PBS group, mice treated with recombinant CXCL3 showed significantly increased proportions of overall neutrophils and TGFβ1+ neutrophils in both the spleen and peripheral blood (Figure S9I–K). Administration of recombinant CXCL3 at graded doses established a dose‑dependent effect on neutrophil expansion and TGFβ1 expression (Figure S9L–M). Concurrent administration of the selective CXCR2 antagonist SB225002 largely abrogated the antitumor effects of CXCL3 antagonism (Figure S9N). SB225002 significantly reduced TGFβ1 expression in recombinant CXCL3‐treated neutrophils in vitro (Figure S9O). For the high burden group, CXCL3 antagonism moderately delayed tumor growth and reduced tumor volume, while also decreasing the proportions of overall neutrophils and TGFβ1+ neutrophils in the spleen and peripheral blood to levels comparable to those in the low burden group (Figure 5F–H). Similarly, in high burden models established with Py8119‑Cxcl3^‑/‑^ cells, tumor size was significantly reduced, accompanied by decreased frequencies of overall and TGFβ1+ neutrophils in the spleen and peripheral blood, and enhanced early activation and long‑term memory phenotypes of CD8+ TRT cells compared with wild‑type controls (Figure 5I,J and Figure S9P). Using Py8119‑Cxcl3^‑/‑^ cells to establish low and high burden models, the difference in rechallenged tumor growth between the two groups was eliminated (Figure S9Q). In vitro, recombinant CXCL3 treatment of neutrophils significantly upregulated their TGFβ1 expression (Figure 5K). In vitro, conditioned media from high burden significantly increased the proportion of TGFβ1+ neutrophils compared with low burden controls, while neutrophil viability remained > 95% in all groups (Figure 5L,M). This effect was abrogated by CXCL3 neutralization in high burden, bringing the TGFβ1+ neutrophil frequency down to levels comparable to those observed with low burden conditioned media (Figure 5M). Treatment of SIINFEKL‑stimulated OT1 CD8+ T cells with conditioned media from neutrophils cultured under low or high burden conditions (N‑CM‑L or N‑CM‑H) revealed that N‑CM‑H significantly attenuated IFNγ expression and CFSE dilution‑indicated cell proliferation compared with N‑CM‑L, both of which were reversed by TGFβ1 neutralization, and a similar trend was observed for the proportion of CD44^hi^CD62L^hi^ cells among OT1 CD8+ T cells (Figure 5N,O). CXCL3 administration did not significantly alter Ki67 expression in SIINFEKL‑stimulated OT‑1 CD8+ T cells compared with control (Figure 5P). In summary, increased CXCL3 release may be the initiating factor through which high tumor burden triggers systemic expansion of immunosuppressive neutrophils and impairment of antitumor immune phenotypes.

### 2.10 CXCL3 Antagonism Rescues Systemic CD8+ TRT Cell Function Impaired by High Tumor Burden

To develop strategies for reversing the impaired immune phenotypes of systemic tumor‐specific CD8+ T cells caused by high tumor burden, we attempted to antagonize CXCL3 in both adjuvant and neoadjuvant settings, specifically during the early phase (Figure 5Q). We established low and high burden models using Py8119 cells expressing ovalbumin (Py8119‐OVA), and employed T‐Select H‐2K^b^ OVA Tetramer‐SIINFEKL (tetramer) staining to detect tumor‐specific CD8+ T cells (Figure 5Q). At day 14 post‐resection, CXCL3 antagonism significantly increased the proportion of tetramer+CD8+ T cells in both the spleen and peripheral blood of the high burden group under adjuvant and neoadjuvant settings compared to the PBS control, restoring it to levels comparable to those in the low burden group (Figure S9R,S). Moreover, CXCL3 antagonism significantly increased the proportions of Ki67+ cells among tetramer+CD8+ T cells in the high burden group (Figure S9T,U). Notably, while CXCL3 antagonism enhanced activation, it also increased the proportion of PD‐1+ cells among tetramer+CD8+ T cells, particularly under the adjuvant setting (Figure S9T,U). However, CXCL3 antagonism did not significantly alter the proportion of Tim3+ cells among tetramer+CD8+ T cells, with comparable levels observed across all groups (Figure S9T,U). Therefore, CXCL3 antagonism in the early phase can reverse the suppression of activation in systemic tumor‐specific CD8+ T cells induced by high tumor burden.

We further assessed whether CXCL3 antagonism modulates the long‐term memory phenotype of systemic tumor‐specific CD8+ T cells. The efficacy of CXCL3 antagonism persisted at day 30 post‐resection, elevating tumor‐specific CD8+ T cell frequencies in the high burden group, regardless of adjuvant or neoadjuvant setting (Figure 5R,S). More importantly, CXCL3 antagonism significantly increased the proportions of CD44^hi^CD62L^hi^ cells and IL7Rα^hi^ cells among tetramer+CD8+ T cells in the high burden group, reaching levels comparable to those in the low burden group (Figure 5T,U). These results demonstrate that early antagonism of CXCL3 is beneficial for reversing the impairment of long‐term memory phenotypes in systemic tumor‐specific CD8+ T cells induced by high tumor burden.

For clinical validation, analysis of the METABRIC database revealed that CXCL3 expression in tumors from T2‑3 breast cancer patients was higher than that in T1 patients, with consistent trends observed in both Luminal and Non‑luminal subgroups (Figure S9V). We performed a pooled analysis of prognostic data from all cancer types in the Cancer Genome Atlas Program (TCGA) database. The results revealed that high expression of CXCL3, as well as a high combined gene signature of CXCL2 and CXCL3, were associated with worse overall survival (OS) and disease‐free survival (DFS) (Figure 5V).

### 2.11 CXCL3 Antagonism Enhances the Antitumor Efficacy of anti‐PD‐1 Therapy

**FIGURE 6:**
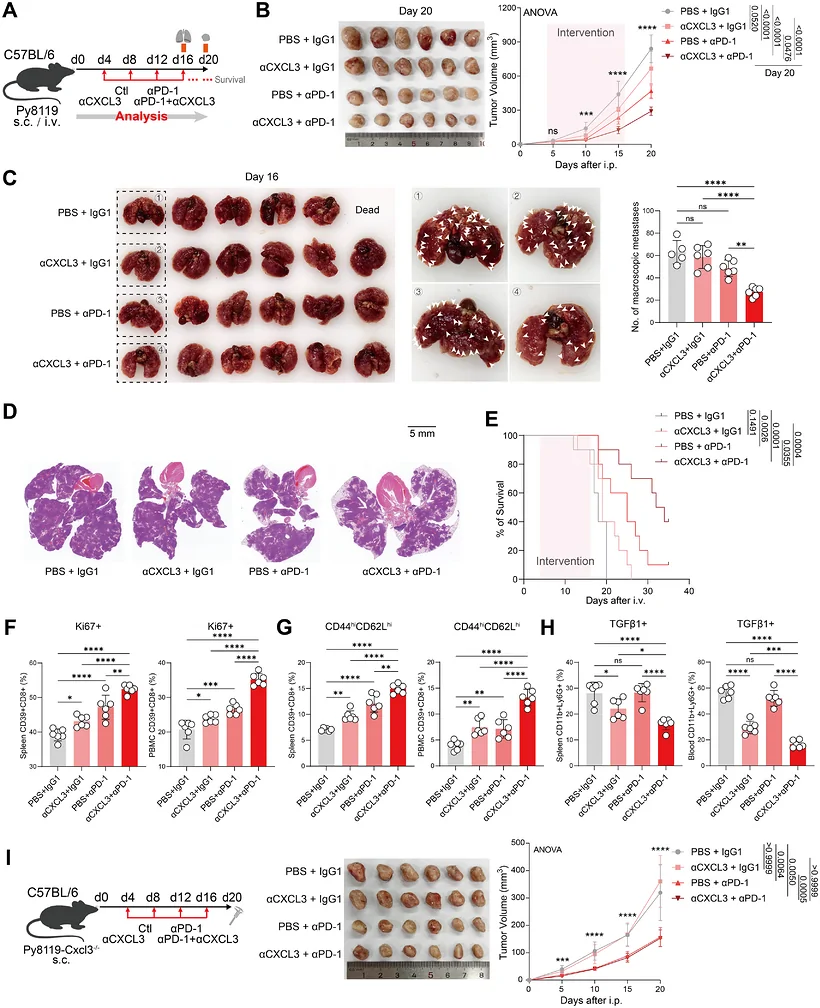
Synergistic antitumor effects of PD‐1 blockade and CXCL3 antagonism in the high burden setting. (A) Experimental design of αPD‐1 and αCXCL3 monotherapy or combination therapy following subcutaneous inoculation or tail vein injection of Py8119 cells under high burden. (B) Tumor growth curves and tumor images in mice treated with PBS + IgG1, αCXCL3 + IgG1, PBS + αPD‐1, or αCXCL3 + αPD‐1 (*n* = 6 per group). (C) Images and bar graph comparing lung metastatic foci numbers after tail vein injection of Py8119 cells from mice treated with PBS + IgG1, αCXCL3 + IgG1, PBS + αPD‐1, or αCXCL3 + αPD‐1 (*n* = 6 per group). White arrows indicate the metastatic lesions. (D) Representative HE‐stained sections of metastatic burden in C). Scale bar: 5 mm. (E) Comparison of survival curves and survival periods in mice treated with PBS + IgG1, αCXCL3 + IgG1, PBS + αPD‐1, or αCXCL3 + αPD‐1 after tail vein injection of Py8119 cells (*n* = 10 per group). (F–H) Comparison of the percentages of Ki67+ cells and CD44^hi^CD62L^hi^ cells among CD39+CD8+ T cells, and TGFβ1+ cells among neutrophils in the spleen and peripheral blood following treatment with PBS + IgG1, αCXCL3 + IgG1, PBS + αPD‐1, or αCXCL3 + αPD‐1 (*n* = 6 per group). (I) Tumor growth curves and tumor images of high burden subcutaneous tumors established with Py8119‐Cxcl3^−/−^ cells following treatment with PBS + IgG1, αCXCL3 + IgG1, PBS + αPD‐1, or αCXCL3 + αPD‐1 (*n* = 6 per group). ns not significant, ^*^ *p* < 0.05, ^**^ *p* < 0.01, ^***^ *p* < 0.001, ^****^ *p* < 0.0001. HE, hematoxylin‐eosin.

Antagonizing CXCL3 to reverse systemic immunosuppression may represent a feasible strategy to enhance immunotherapy efficacy. Moreover, our previous findings indicate that CXCL3 antagonism increases PD‑1 expression on tumor‑specific CD8+ T cells (Figure S9T,U). We therefore investigated the synergistic effects of combining CXCL3 antagonism with anti‐PD‐1 therapy. Subcutaneous or tail vein injection of Py8119 cells was performed to establish orthotopic and pulmonary metastasis models with high tumor burden, respectively (Figure 6A). Monotherapy with either CXCL3 antagonism or anti‐PD‐1 monotherapy moderately slowed the growth of subcutaneous tumors, whereas combining CXCL3 antagonism with anti‐PD‐1 therapy further suppressed tumor progression and significantly reduced tumor volume by day 20 (Figure 6B). In the tail vein injection model, neither CXCL3 antagonism nor anti‑PD‑1 monotherapy significantly reduced pulmonary metastatic burden, whereas their combination did (Figure 6C,D). CXCL3 antagonism alone failed to significantly improve mouse survival, whereas anti‐PD‐1 monotherapy significantly improved survival; the combination therapy further increased overall survival significantly (Figure 6E). Compared with monotherapy, the combination further increased the proportions of Ki67+ and CD44^hi^CD62L^hi^ cells among CD39+CD8+ T cells, and further reduced TGFβ1+ neutrophil frequencies in both spleen and peripheral blood (Figure 6F–H). In high burden subcutaneous tumor models established with Py8119‑Cxcl3^‑/‑^ cells, the synergistic effect of CXCL3 antagonism and anti‑PD‑1 therapy was abrogated (Figure 6I). Therefore, CXCL3 antagonism can enhance the systemic antitumor efficacy of anti‐PD‐1 therapy.

## 3 Discussion

The occurrence and progression of malignant tumors can lead to the formation of a complex, interconnected immune‐suppressive network in organs such as the bone marrow, blood, and spleen [11, 42, 43]. Factors involved in this network include the increase in MDSCs, the expansion of suppressive lymphocyte subpopulations, reduced TCR diversity, and impaired quantity and function of cytotoxic lymphocytes [9, 42, 44, 45, 46, 47]. In addition, tumor‐derived suppressive cytokines, such as IL10 and TGFβ, also play an important role [43, 48]. In our study, analysis of public single‐cell data revealed that highly malignant tumors exert a greater impact on the systemic antitumor immune status, particularly on CD8+ T cells. The disruption of systemic immunity by tumors provides favorable conditions for recurrence and metastasis, which cannot be overlooked in clinical treatment.

Unlike early‐stage tumors, advanced‐stage tumors often exhibit inferior responses to immunotherapy, such as ICB [5, 49, 50]. Besides, therapeutic responses to ICB in advanced‐stage tumor patients are often limited to those with high PD‐L1 expression in tumors [6, 7, 8]. Conventional tumor immunology research has focused on immune cell function within the tumor microenvironment; however, the effective implementation of local antitumor immunity requires the involvement of systemic immunity [51, 52, 53]. Whether alterations in systemic antitumor immune status are associated with tumor burden has not been fully established. Our work shifts the focus from a sole emphasis on the local tumor microenvironment to a perspective of the holistic systemic macroenvironment. Our study provides a conceptual advance by dynamically linking the tumor burden to the long‐term immune memory of the systemic immune compartment, revealing that tumors remotely and persistently regulate host immunity. This extends our understanding of how tumor burden shapes systemic immunity beyond the local microenvironment [54, 55]. First, using both clinical data and murine models, we demonstrated that high tumor burden significantly impairs the activation and memory phenotypes of CD8+ T cells, which persists even after complete tumor resection. Furthermore, scRNA/TCR‐seq analysis revealed that Cxcr6+Cd8+ TRT cells represent a key subset mediating systemic antitumor immune memory. This subset characteristically exhibits high expression of Cxcr6 and Entpd1 (CD39). In previous studies, CXCR6+CD8+ T cells have been identified as a population with specific antitumor effector functions and long‐term protective memory, and are closely associated with the efficacy of ICB therapy [56, 57, 58]. While CD8 depletion confirmed the overall CD8+ T cell dependence of this memory defect, the subset‐specific involvement of CXCR6+CD8+ TRT cells was further defined by CXCR6 neutralization and adoptive transfer experiments. In our study, functional analysis revealed that compared to the low burden group, Cxcr6+CD8+ TRT cells in the high burden group exhibited impaired activation and proliferation in the early phase, leading to a weaker long‐term memory phenotype. Besides, high tumor burden compromised the maintenance of both tumor reactivity and memory in Cxcr6+CD8+ TRT cells. The dynamic changes of this subset can serve as a core phenotype connecting high tumor burden to systemic antitumor immune dysfunction, and can be utilized as a potential cellular biomarker for assessing systemic antitumor immune status. More importantly, this divergence in systemic immunity helps explain the diminished efficacy of immunotherapy in advanced settings and underscores the necessity for strategies that specifically target the systemic immunosuppression unique to high burden tumors.

While total CD8+ T cell frequencies are often linked to immunotherapy responses, this association is not absolute, as some tumors with progenitor exhausted T cell infiltrates may still respond to anti‐PD‑1 therapy [59]. Our findings do not merely reflect reduced CD8+ numbers. Instead, we identify a specific defect in the CXCR6+CD8+ TRT subset, a population with high tumor reactivity and memory potential, as the key lesion induced by high burden. This functional impairment persisted even after total CD8+ counts recovered. Notably, CD8+ TRT cells did not show significant upregulation of exhaustion markers across groups, suggesting that the defect is not classical exhaustion but rather an impairment in early activation and memory programming.

CXCL3 is expressed in multiple tumors, including pancreatic ductal adenocarcinoma, colorectal cancer, and thyroid carcinoma, and its expression is associated with poorer treatment response and clinical prognosis [60, 61, 62]. Tumor‐derived CXCL3 binds to CXCR2 on MDSCs, such as neutrophils, promoting their migration into the tumor microenvironment [63]. However, we found that tumor‐derived CXCL3 also induces sustained recruitment of neutrophils in peripheral tissues, such as the spleen and peripheral blood, which has not been previously reported. Neutrophils are functionally plastic, with anti‑tumor (N1‑like) and pro‑tumor (N2‑like) states shaped by the local microenvironment [64]; in our model, CXCL3 signaling drives the suppressive N2‑like polarization. Specifically, compared to low burden tumors, high burden tumors release more CXCL3 into the periphery via the JAK‐STAT3 pathway. We do not deny that tumor‑intrinsic factors beyond size, such as genetic heterogeneity, metabolic profile, and stromal composition, may influence the engagement of this axis. CXCL3 binds to CXCR2 on systemic neutrophils, activating their immunosuppressive phenotype and enhancing the self‐recruitment and positive feedback loop of TGFβ1+ neutrophils, ultimately impairing the effector and memory functions of CD8+ TRT cells. This effect can be reversed by neutrophil depletion in the early phase. The precise temporal precedence between neutrophil expansion and CD8+ TRT cell dysfunction will require further investigation. Although chemotherapy drugs such as paclitaxel can reduce systemic neutrophils, it fails to effectively reverse, and may even exacerbate, immune impairment due to their concomitant damage to lymphocytes [35, 36]. However, given paclitaxel's broad cytotoxicity, its failure does not diminish the specificity of the anti‑Ly6G findings. Besides, whether the CXCL3‑neutrophil‑TGFβ1 axis also actively suppresses the production of T cell‑recruiting chemokines such as CXCL9 and CXCL10 remains to be determined and represents an intriguing direction for future investigation. Whether the tumor‐free dose‐response fully reflects the altered neutrophil state in tumor‐bearing hosts also merits consideration. Of note, CXCL3 expression in breast cancer is more complex and context‐dependent than in other tumor types. This complexity may reflect molecular subtype heterogeneity or tissue‑specific differences in CXCL3 regulation. We acknowledge that although macrophage depletion failed to rescue CD8+ TRT cell phenotypes, macrophages may still contribute to the immunosuppressive milieu in ways not captured by the current experimental design. Accordingly, targeted neutralization of neutrophils represents one of the strategies to improve systemic antitumor immune memory in tumor models.

In distinction from neutrophil depletion, targeting CXCL3 represents a more clinically translatable therapeutic strategy. Previous studies have shown that low expression of CXCL3 in colorectal cancer is associated with improved sensitivity to immunotherapy [63]. In this study, we demonstrated that systemic CXCL3 antagonism reduces systemic TGFβ1+ neutrophil accumulation and partially suppresses local progression of high burden tumors. Neutrophils are functionally plastic and can adopt anti‑tumor (N1‑like) or pro‑tumor (N2‑like) phenotypes depending on the local microenvironment [65]. In our model, we speculated that sustained systemic CXCL3 signaling, together with TGFβ1‑rich conditions under high tumor burden, drives polarization toward the suppressive N2‑like state, whereas low burden settings may favor a less polarized or protective phenotype. Given the critical role of early neutrophil recruitment in shaping subsequent antitumor immunity, we administered CXCL3 antagonism in both low and high burden groups under adjuvant or neoadjuvant settings (i.e., within 10 days post‐ or pre‐resection). We found that, regardless of the treatment setting, CXCL3 antagonism restored the early activation, proliferation, and long‐term memory phenotypes of systemic tumor‐specific CD8+ T cells in the high burden group to levels comparable to the low burden group, without affecting the expression of exhaustion markers. Currently, ICB plays a critical role in the systemic treatment of malignant tumors [66, 67, 68]. Nevertheless, despite treatment with ICB, a significant proportion of patients still fail to achieve satisfactory therapeutic outcomes [69, 70, 71, 72]. Previous studies have suggested that the tumor reactivity, activation, and memory phenotype of T cells are critical for the efficacy of ICB [73, 74, 75, 76, 77, 78]. Moreover, the systemic immune status is closely associated with the efficacy of ICB therapy [79, 80, 81]. The modest efficacy of anti‐PD‐1 monotherapy in the high burden model mirrors the poor prognosis and checkpoint resistance of advanced breast cancer, underscoring the necessity of counteracting CXCL3‐driven systemic immunosuppression to enable effective immunotherapy. In extended investigations, we validated the synergistic effects of CXCL3 antagonism combined with anti‐PD‐1 therapy, observed in both orthotopic and metastatic tumor models. Thus, CXCL3 antagonism repairs the systemic antitumor immune “scarring” caused by high tumor burden, restores long‐term immune memory, and represents a potential combinatorial target for enhancing the efficacy of ICB therapy. Timely systemic CXCL3 antagonism holds significant translational significance for improving the efficacy and prognosis of patients with high tumor burden. Although the data from human peripheral blood mononuclear cells (PBMCs) were derived from treatment‑naïve patients and do not directly link to anti‑PD‑1 responses, we believe that characterizing baseline systemic immunity across tumor burdens is a necessary first step toward understanding ICB resistance. The mouse combination therapy experiments serve as a preclinical proof‑of‑concept, and direct clinical validation of the burden‑dependent immune impairment in predicting ICB outcomes remains to be established.

Our study has several limitations. The analysis was primarily based on two tumor‐bearing mouse models (Py8119 and MC38) and partial clinical data, and has not yet been validated in additional cancer types or clinical cohorts. The findings regarding CXCL3 antagonism are mainly derived from the Py8119 model, which we selected due to its more pronounced systemic effects. Besides, whether CXCL3 influences other immune cells, such as Tregs or other MDSC subsets, requires further investigation. While neutrophils are a major source of TGFβ1 in our models, we cannot exclude contributions from other lineages, and the exclusive role of neutrophils in mediating this immunosuppressive effect warrants further investigation. Moreover, we mainly focused on phenotypic observations and functional validation; the exploration of specific downstream molecular regulatory mechanisms, such as the downstream signaling pathways by which CXCL3 induces TGFβ1 in neutrophils and TGFβ1 suppresses CD8+ TRT cells, remains incompletely defined. Whether TGFβ1 neutralization alone recapitulates the in vivo effects of CXCL3 antagonism remains to be determined. The full functional phenotype and additional suppressive mechanisms of burden‐induced neutrophils beyond TGFβ1 remain to be characterized. Furthermore, while CXCL3 antagonism showed promising efficacy in our model, we cannot rule out the possibility that prolonged blockade of this pathway may lead to compensatory upregulation of other immunosuppressive chemokines or pathways, as has been observed with sustained targeting of the CXCR2 signaling axis [82]. Finally, the human PBMC data are exploratory, and larger, prospective burden‐stratified cohorts with linked systemic immune and treatment‐response measurements are required.

## 4 Conclusion

Our study demonstrates that high tumor burden suppresses systemic CD8+ T cell‐induced antitumor immune memory via the CXCL3‐neutrophil‐TGFβ1 axis. The CXCL3 antagonism strategy effectively reverses this immunosuppressed status and synergizes with anti‐PD‐1 therapy. These findings provide a potential, novel approach to combination immunotherapy for patients with high tumor burden, particularly those with locally advanced or advanced disease. The clinical relevance of the CXCL3‐neutrophil‐TGFβ1 axis in other cancers remains to be established.

## 5 Experimental Section

### 5.1 Study Approval

All the mice were purchased from GemPharmatech (Nanjing, China) and were housed under specific pathogen‐free conditions in the Animal Core Facility of Nanjing Medical University. The dark/light cycle was 12 h. The drinking water for the mice was acidified to a pH of 2.5–3.0. The mice were fed cobalt‐60‐irradiated, sterilized feed. All the mice used in this study were female. The mouse experiments were approved by the Institutional Animal Care and Use Committee (IACUC), Animal Core Facility of Nanjing Medical University (IACUC‐2507059).

### 5.2 Cell Lines

The C57BL/6 mice‐derived mammary carcinoma Py8119 cell line was purchased from the American Type Culture Collection (USA), and the C57BL/6 mice‐derived colon adenocarcinoma cell line MC38 was purchased from Kerafast (USA). The Py8119 cell line was cultured in Ham's F‐12K (G4560, Servicebio, China) supplemented with 10% fetal bovine serum (086‐150, WISENT, Canada), 100 units/mL penicillin, 100 µg/mL streptomycin (BL505A, Biosharp, China), 0.1% mycoplasma elimination reagent (BL591A, Biosharp, China). The MC38 cell line was cultured in RPMI1640 (350‐006‐CL, WISENT, Canada) supplemented with the aforementioned additives. All cells were cultured under conditions of 37°C and 5% CO_2_. Py8119 served as the primary model, whereas MC38 was included as an independent model to assess whether the burden‐associated phenotype extended across tumor types.

### 5.3 Mouse Tumor Model

To avoid the impact of distant metastasis on the results, we selected the Py8119 and MC38 cell lines for orthotopic inoculation, as they are relatively less likely to form substantial distant metastatic foci, while maintaining relatively high malignancy. On day 0, Py8119 or MC38 cells were inoculated subcutaneously into the right fourth mammary fat pad of 6 week‐old female mice at a density of 5 × 10^5^ cells (low burden group) or 3 × 10^6^ cells (high burden group) in 100 µL phosphate buffer saline (PBS) (G4202, Servicebio, China). MC38 cells were inoculated subcutaneously into the right inguinal flank, at the same density as mentioned above. No metastasis was observed in mice inoculated with tumor cells on day 14, indicating that the tumor was eligible for surgical resection. For rechallenge, 5 × 10^5^ Py8119 or MC38 cells were inoculated subcutaneously on the left fourth mammary fat pad or the left inguinal flank. For the tail vein rechallenge following resection, 1 × 10^5^ Py8119 cells were injected. In the investigation of synergistic effects between CXCL3 antagonism and anti‐PD‐1 therapy, Py8119 cells were inoculated subcutaneously (3 × 10^6^ cells) or intravenously via the tail vein (4 × 10^5^ cells), in order to maintain a high tumor burden. For challenge following adoptive transfer of CXCR6+CD8+ TRT cells into naïve recipients, mice were subcutaneously inoculated with 5 × 10^5^ Py8119 cells. Tumor length and width were measured using a digital vernier caliper, and tumor volume was calculated using the formula: volume = (length × width (2))/2. The MC38 model was included alongside Py8119 to assess whether the burden‑dependent systemic immune phenotypes are generalizable across tumor types with distinct tissue origins and immunogenicity profiles.

### 5.4 Mouse Tumor Resection

After establishing the low burden and high burden models, mice were anesthetized using isoflurane (R510‐22‐10, RWD, China) via an anesthesia gas machine. The mice were secured on the operating table in a supine position, and the surgical site was disinfected with iodophor disinfectant. A sterile surgical scissor was used to make an incision around the tumor, ensuring the incision was large enough to fully expose the tumor. We maintained approximately consistent incision lengths between low and high burden groups. The tumor was carefully dissected from surrounding tissues using forceps and surgical scissors, avoiding direct contact with the tumor and preventing damage to blood vessels and vital organs. The tumor was completely resected to ensure no residual tumor tissue remained. During the surgery, the draining lymph node on the operative side was preserved. The surgical area was rinsed with saline to remove blood and tissue debris. The incision was closed with interrupted sutures and reinforced using wound clips designed for mice. The length of surgical incisions was measured using a sterilized steel ruler. Then, the incision was disinfected again with iodophor disinfectant. Postoperatively, the mice were monitored regularly to ensure proper wound healing and to check for any signs of infection.

### 5.5 Hematological Analysis

After inducing anesthesia in mice via isoflurane inhalation, an appropriate volume of peripheral blood was collected from one eyeball into an EDTA‐anticoagulant tube. Following collection, the tube was gently inverted several times to ensure thorough mixing of the blood with EDTA. Automated hematology analysis was performed using a fully automatic blood cell analyzer (BC‐2800 Vet, Mindray Animal Medical Technology, China). The primary parameters evaluated included white blood cell count, neutrophil percentage, monocyte percentage, and lymphocyte percentage.

### 5.6 Serum Isolation from Mouse Peripheral Blood

Peripheral blood samples collected from mice were allowed to clot at room temperature for 2 h, followed by centrifugation at 3000 rpm for 15 min at 4°C. The supernatant (serum) was then carefully aspirated and stored for subsequent analysis.

### 5.7 Enzyme‐Linked Immunosorbent Assay (ELISA)

The samples were carefully collected and analyzed using pre‐coated ELISA kits according to the manufacturers’ protocols. Briefly, 100 µL of samples, standards, and blank controls were added to the plate and incubated at 37°C for 1–2 h. After washing (3–5 times), biotinylated antibody working solution was added and incubated for 1 h, followed by washing and addition of enzyme‐conjugated secondary antibody with 30 min light‐protected incubation. The 3,3’,5,5’‐Tetramethylbenzidine substrate was then added for color development, the reaction was stopped with 2M sulfuric acid, and absorbance was measured at 450 nm. Sample concentrations were calculated based on the standard curve. The ELISA kits used in this study were as follows: Mouse C‐Reactive Protein/CRP ELISA Kit (EK294/2, Multi Sciences, China), Mouse CXCL3 ELISA Kit (MM‐44986M1, MEIMIAN, China), Mouse CXCL2 ELISA Kit (MM‐1012M1, MEIMIAN, China).

### 5.8 Quantification of AST and ALT Concentrations in Mouse Serum

Serum samples were collected from mice using the aforementioned method. The concentrations of AST and ALT in peripheral blood were automatically quantified using a full‐automatic biochemistry analyzer (Chemray‐240, Rayto, China). Following the assay, the results were exported directly from the analyzer for subsequent statistical analysis.

### 5.9 Immunohistochemistry (IHC) Staining

IHC was performed using the Enhance Labelled Polymer System. FFPE tissue sections were deparaffinized in xylene, rehydrated, and treated with 3% hydrogen peroxide to block endogenous peroxidase activity. Antigen retrieval was carried out in Tris‑EDTA buffer (pH 9.0) using a heating method. Non‑specific binding was blocked with 5% BSA for 20 min at room temperature. Sections were then incubated with anti‐CD8 alpha antibody (ab217344, Abcam, UK; 1:1000) overnight at 4°C, followed by HRP‑conjugated secondary antibody from the Dako REAL EnVision Detection System (K5007, DAKO, Denmark) for 30 min at 37°C. Signals were developed with DAB substrate, and sections were counterstained with hematoxylin, dehydrated, cleared, and mounted. Images were acquired, and the average optical density was quantified using ImageJ software (version 1.54g).

### 5.10 Multiplex Immunofluorescence (mIF) Staining

Paraffin sections were dewaxed in an environmentally friendly dewaxing solution (G1128, Servicebio, China) and rehydrated. Antigen retrieval was performed in sodium citrate buffer (pH 6.0) (G1202, Servicebio, China) using microwave heating. Endogenous peroxidase was blocked with 3% hydrogen peroxide, and non‑specific binding was blocked with 3% BSA (GC305006, Servicebio, China). Primary antibodies were applied overnight at 4°C, followed by HRP‑conjugated or fluorescent secondary antibodies at room temperature in the dark. For HRP‑conjugated secondary antibodies, tyramide signal amplification (TSA) fluorophores (G1233/G1231/G1232/G1250, Servicebio, China) were used for signal development, and sections were washed with TBST (G0004, Servicebio, China). Subsequent rounds of staining were performed following the same procedure. Nuclei were counterstained with DAPI (G1012, Servicebio, China), and autofluorescence was quenched before mounting with antifade mounting medium (G1401, Servicebio, China). Images were acquired using a digital slide scanner (Pannoramic MIDI, 3DHISTECH, Hungary) and analyzed with CaseViewer software (version 2.4). Results are presented as absolute counts per field.

The primary antibodies used in IF were as follows: Recombinant Anti‐CD8 alpha antibody (GB15068, Servicebio, China), Anti‐CD39 Rabbit pAb (GB111582, Servicebio, China), Anti‐CD44 Rabbit pAb (GB112054, Servicebio, China), CD62L Recombinant Rabbit Monoclonal Antibody (MA5‐29789, eBioscience, Thermo Fisher Scientific, USA), Anti‐Ly6g Rabbit pAb (GB11229, Servicebio, China), Anti‐TGF beta 1 Rabbit pAb (GB11179, Servicebio, China), Anti‐F4/80 Rat mAb (GB12027, Servicebio, China). The secondary antibodies used in IF were as follows: HRP‐conjugated Goat Anti‐Rabbit IgG (GB23303, Servicebio, China), S‑vision immunohistochemical polymer secondary antibody (goat anti‑rabbit) (G1302, Servicebio, China), HRP conjugated Goat Anti‐Rat IgG (H+L) (GB23302, Servicebio, China), Alexa Fluor 488‐conjugated Goat Anti‐Rabbit IgG (GB25303, Servicebio, China).

### 5.11 Hematoxylin‐Eosin (HE) Staining

The paraffin‐embedded sections were baked at 60°C and then deparaffinized in xylene (10023418, Sinopharm Chemical Reagent, China) and rehydrated through a graded ethanol series. Subsequently, the sections were stained with hematoxylin (H3136, Sigma, Germany), followed by rinsing in running water. Differentiation was performed using 0.7% acid alcohol, and bluing was achieved under running water. The sections were then dehydrated in 95% ethanol and counterstained with eosin (E4009, Sigma, Germany). Further dehydration was carried out in graded ethanol and xylene solutions. Finally, the sections were mounted with neutral balsam (10004160, Sinopharm Chemical Reagent, China).

### 5.12 Preparation of the Single‐Cell Suspension of Mouse Spleen

After euthanizing the mice, the spleen was dissected and separated. The obtained spleen tissue was gently pressed and triturated using a plunger of a 10 mL syringe, and the resulting suspension was filtered through a 70 µm nylon mesh cell strainer (BS‐70‐CS, Biosharp, China). After red blood cells were removed using an erythrocyte‐lysing reagent (BL503B, Biosharp, China), the cells were washed with PBS, resuspended, and counted for subsequent experiments.

### 5.13 Isolation of PBMCs from Mice

Depending on the experimental needs, peripheral blood samples from mice were obtained through eye enucleation and collected into EDTA‐K2 tubes. The PBMCs were isolated via density gradient centrifugation and resuspended in PBS for subsequent analysis. For the neutrophil‐containing flow cytometry panel, whole blood samples were treated with erythrocyte‐lysing reagent (BL503B, Biosharp, China), followed by washing steps to generate single‐cell suspensions for subsequent antibody staining.

### 5.14 Isolation of Bone Marrow Cells From Mice

Bone marrow cells were flushed from the femurs of euthanized mice with PBS using a 27G needle, filtered through a 70 µm cell strainer, and centrifuged. Red blood cells were lysed with erythrocyte‐lysing reagent. After washing, cells were resuspended in PBS or flow cytometry staining buffer for downstream applications.

### 5.15 Analysis of scRNA‐Seq Data From Human PBMC Samples

In this study, we utilized scRNA‐seq data from several clinical studies, including GSE231794 [24], GSE274381 [25], and data from two previously conducted clinical trials (NCT04805736; ChiCTR2000029155) [23, 26] by our team. Ultimately, PBMC samples from 4 healthy controls, 10 non‐TNBC patients, and 3 TNBC patients were included. The latter two groups consisted of newly diagnosed, treatment‐naïve breast cancer patients without distant metastasis, representing baseline conditions. Briefly, we performed data integration, quality control, batch effect removal, dimensionality reduction, clustering, cell type annotation, gene set scoring, and visualization on the raw data.

### 5.16 Dissection of Spleen Tissue and Preparation of Single‐Cell Suspension

For each condition and time point, spleen samples from five biologically independent mice were pooled prior to library preparation to minimize inter‑individual variability. The pooled spleen tissues were preserved in sCelLive Tissue Preservation Solution (1010012, Singleron, China) at 4°C after mouse euthanasia and dissection under sterile conditions within 5 min. The samples were then rinsed three times with Hanks' balanced salt solution and finely minced. Enzymatic digestion was performed using 3 mL of sCelLive Tissue Dissociation Solution (1020012, Singleron, China) in conjunction with the Singleron PythoN Tissue Dissociation System at 37°C for 15 min. The resulting cell suspension was passed through a 40‐micron sterile strainer, and red blood cells were lysed by mixing with GEXSCOPE Red Blood Cell Lysis Buffer (RCLB) (Singleron, China) at a 1:2 ratio (cell suspension to RCLB). This mixture was incubated at room temperature for 5–8 min. Following centrifugation at 300×g for 5 min at 4°C, the supernatant was discarded, and the cell pellet was resuspended in PBS. Finally, cell viability was evaluated microscopically after staining with 0.4% Trypan Blue.

### 5.17 Library Preparation for scRNA‐Seq and scTCR‐Seq

A single‐cell suspension was loaded onto a 10× Chromium A Chip, and scRNA‐seq libraries were prepared according to the 10× Genomics Chromium Single Cell Immune Profiling Solution protocol. In summary, approximately 20 000 cells with 90%–95% viability were encapsulated into droplets at a density of 900 cells/µL. Following reverse transcription, the droplets were disrupted, and the barcoded cDNA was purified using DynaBeads, after which it underwent polymerase chain reaction (PCR) amplification. Partial cDNA fragments were processed and spliced to construct sequencing libraries compatible with the Illumina platform for single‐cell transcriptome analysis. The remaining cDNA was further enriched to generate TCR libraries. These enriched products were then amplified using the Chromium Single Cell V(D)J Enrichment Kit to create sequencing libraries suitable for the Illumina platform. Finally, each library was sequenced on the Illumina NovaSeq 6000 system with 150 bp paired‐end reads. The target sequencing depth was set at an average of 30 000 read pairs per cell for the mRNA library, and 3000 read pairs per cell for the TCR libraries.

### 5.18 Analysis and Processing of scRNA‐Seq and scTCR‐Seq Data

The raw data of scRNA‐seq were initially transformed into fastq format utilizing the Illumina bcl2fastq conversion tool, followed by filtration to yield high‐quality clean data. This filtration process adhered to specific criteria: (1) elimination of polyA sequences, (2) exclusion of reads with over three ambiguous bases, and (3) discarding of reads of inferior quality (where bases with a Q score of 5 or below constituted more than 20% of the total reads). Subsequently, the refined data were processed with the CellRanger software (version 7.0.0) from 10X Genomics to demultiplex cell barcodes and align valid barcodes, while STAR facilitated the alignment of these reads to the reference genome (mm10‐2020‐A). The quantification of gene expression was conducted by enumerating unique molecular identifiers (UMIs) through the CellRanger software.

The scRNA‐seq data were analyzed using the Seurat package (version 4.4.0) in R software (version 4.3.0). In quality control, low‐quality cells were removed. The criteria for identifying low‐quality cells were: fewer than 250 genes or more than 2500 genes, fewer than 500 UMIs, mitochondrial gene content greater than 15%, and potential doublets. After filtering, a total of 56 203 cells were used for subsequent analysis. Data normalization was performed based on the raw UMIs counts of each cell. The FindVariableFeatures function was used to identify 2000 highly variable genes. These genes were scaled using the ScaleData function, followed by dimensionality reduction via principal component analysis. Batch correction was performed on the merged sample data using the Harmony package (version 1.2.0). The FindNeighbors function was used to calculate shared nearest neighbor similarity in the batch‐corrected cell‐PC matrix. Cell clusters were identified using the FindClusters function, which optimizes the clustering algorithm through the Shared Nearest Neighbor module (dims.use = 1:30, resolutio*n* = 1.0). The cell clustering was visualized through Uniform Manifold Approximation and Projection (UMAP). The differential gene expression between cell clusters was analyzed using the COSG package (version 0.9.0) [83] or FindAllMarkers function in Seurat, and cell types were manually annotated based on marker genes of each cluster. Gene set scores were calculated using the AUCell package (version 1.24.0) or AddModuleScore function in Seurat. Gene sets for immune cell functional scoring were obtained from the Kyoto Encyclopedia of Genes and Genomes (KEGG) (https://www.kegg.jp/) and MSigDB (http://www.gsea‐msigdb.org/) databases. The pseudotime analysis was performed using the Monocle package (version 2.30.1), while cell‐cell communication analysis was conducted using the CellChat package (version 1.6.1). The analysis results were mainly visualized using the ggplot2 package (version 3.5.1).

The assignment of TCR clonotypes was conducted using the cellranger vdj pipeline (version 7.1.0), with vdj_GRCm38_alts_ensembl‐7.0.0 as reference. Through this method, a TCR diversity matric containing clonotype frequency and barcode information was obtained. For single‐cell TCR data, only cells with one productive TCR α‐chain (TRA) and one productive TCR β‐chain (TRB) were included and analyzed. Each unique TRA‐TRB pair was defined as a clonotype. If at least two T cells shared the same TCR clonotype, these cells were defined as clonal T cells. The number of T cells harboring a specific TCR clonotype represents the degree of clonality.

### 5.19 Library Construction for Bulk RNA‐Seq of Cell and Tissue Samples

In cell sample preparation, 1 × 10^6^ or 8 × 10^6^ Py8119 cells were plated in 100 mm culture dishes and cultured for 48 h to obtain relatively sparse or dense cell populations, simulating low and high tumor burden, respectively, in vitro. Total RNA was extracted from cell samples using Trizol reagent (15596026, Thermo Fisher Scientific, USA), followed by rapid freezing in liquid nitrogen and storage at −80°C. For tissue sample preparation, established mice with low or high Py8119 tumor burden were euthanized, and tumor tissues were aseptically dissected. Each tumor sample was cut into approximately 0.5 cm × 0.5 cm piece, then placed in specialized RNA tissue preservation solution (LC‐RNS‐005, LC Sciences, China), subjected to gradient cooling, and ultimately stored at −80°C.

Total RNA quality and concentration were assessed using an Agilent Bioanalyzer 2100 with the RNA 6000 Nano LabChip Kit (5067‐1511, Agilent, USA). Only samples with RNA integrity numbers > 7.0 were processed for downstream analysis. Poly(A) mRNA was isolated from 1 µg total RNA through two rounds of purification with Dynabeads Oligo (dT) magnetic beads (61005, Thermo Fisher, USA). The purified mRNA was then fragmented by heat‐activated divalent cation cleavage. Following the manufacturer's instructions for the Illumina mRNA‐seq sample preparation kit (Illumina, USA), the fragmented RNA was reverse‐transcribed to construct the final cDNA library, which showed an average insert size of 300 bp (± 50 bp). Finally, paired‐end sequencing (2 × 150 bp, PE150) was performed on an Illumina Novaseq 6000 platform (LC‐Bio Technology Co., Ltd., Hangzhou, China) according to the manufacturer's standard protocol.

### 5.20 Analysis of Bulk RNA‐Seq Data

Quality control was performed using fastp (version 1.0.1, https://github.com/OpenGene/fastp) for raw sequence verification. Clean reads were then aligned to the Mus musculus genome (GRCm39) using HISAT2 (version 2.2.1, https://daehwankimlab.github.io/hisat2/) with default parameters. Transcript assembly for each sample was conducted with StringTie (version 3.0.0, https://ccb.jhu.edu/software/stringtie/), followed by expression quantification of all annotated transcripts using both StringTie and ballgown (version 2.40.0, https://www.bioconductor.org/packages/release/bioc/html/ballgown.html). Gene expression levels were normalized and reported as fragments per kilobase of transcript per million mapped reads (FPKM) values to enable cross‐sample comparison of mRNA abundance. The differentially expressed genes were identified using the limma package (version 3.64.1, https://www.bioconductor.org/packages/release/bioc/html/limma.html).

### 5.21 Analysis of TCGA and METABRIC Database

In the comparative analysis of OS and DFS using the TCGA dataset, the GEPIA2 web tool [84] (http://gepia2.cancer‐pku.cn/) was employed with the group cutoff set at the median. Differential expression of CXCL3 in tumor tissues from breast cancer patients across different T stages was analyzed using R software (version 4.3.0) in the METABRIC cohort.

### 5.22 Flow Cytometry

Flow cytometry and corresponding analysis of immune cell composition and function were performed using isolated spleen cells or peripheral blood immune cells from mice. Resuspended cells were stained with Zombie Aqua Fixable Viability Kit (423101/423102, BioLegend, USA) for 20 min at 25°C in 100 µL of PBS. Cell surface antigens were stained with fluorescence‐conjugated antibodies for 30 min at 4°C. For intracellular and nuclear staining, cells that were already surface‐stained were first fixed and permeabilized using fixation/permeabilization buffer according to the manufacturer's instructions (IC001, Multi Sciences, China). Then, the permeabilized cells were incubated with fluorescence‐conjugated antibodies in the permeabilization buffer. Flow cytometry data were obtained using the CytoFLEX LX (Beckman Coulter, USA), CytoFLEX S (Beckman Coulter, USA), or BD FACSCanto System (USA), and analyzed with FlowJo software (version 10.8.1, USA). Gating strategies for flow cytometry are shown in Figure S10. Intracellular TGFβ1 staining was performed without additional ex vivo stimulation.

The fluorescence‐conjugated antibodies used for flow cytometry in this study were as follows: PerCP/Cyanine5.5 anti‐mouse CD45 Antibody (103131, BioLegend, USA), FITC anti‐mouse CD45 Antibody (103108, BioLegend, USA), Pacific Blue anti‐mouse CD45 Antibody (103126, BioLegend, USA), APC/Cyanine7 anti‐mouse CD3 Antibody (100222, BioLegend, USA), APC anti‐mouse CD8a Antibody (162305, BioLegend, USA), APC CD8a Monoclonal Antibody (17‐0081‐82, eBioscience, Thermo Fisher Scientific, USA), PE anti‐mouse/human CD44 Antibody (103007, BioLegend, USA), Alexa Fluor 700 anti‐mouse/human CD44 Antibody (103025, BioLegend, USA), PE/Cyanine7 anti‐mouse CD62L Antibody (104417/104418, BioLegend, USA), PE anti‐mouse/human CD11b (101207, BioLegend, USA), Brilliant Violet 605 anti‐mouse/human CD11b Antibody (101257, BioLegend, USA), PE/Cyanine7 anti‐mouse/human CD11b Antibody (101215, BioLegend, USA), Alexa Fluor 700 IFN gamma Monoclonal Antibody (56‐7311‐82, eBioscience, Thermo Fisher Scientific, USA), Alexa Fluor 488 Ki‐67 Monoclonal Antibody (53‐5698‐82, eBioscience, Thermo Fisher Scientific, USA), PE CD39 Monoclonal Antibody (12‐0391‐80, eBioscience, Thermo Fisher Scientific, USA), PE LAP (TGFβ1) Monoclonal Antibody (12‐9821‐82, eBioscience, Thermo Fisher Scientific, USA), PE/Cyanine7 anti‐mouse Ly‐6G Antibody (127617, BioLegend, USA), FITC anti‐mouse Ly‐6G Antibody (127605, BioLegend, USA), APC anti‐mouse Ly‐6G Antibody (127613, BioLegend, USA), PerCP/Cyanine5.5 anti‐mouse CD127 (IL‐7Rα) Antibody (135021, BioLegend, USA), T‐Select H‐2Kb OVA Tetramer‐SIINFEKL‐PE (TS‐5001‐1C, MBL, Japan), Pacific BlueTM anti‐mouse Lineage Cocktail with Isotype Ctrl (133305, BioLegend, USA), PerCP/Cyanine5.5 anti‐mouse CD117 (c‐kit) Antibody (105823, BioLegend, USA), PE/Cyanine7 anti‐mouse Ly‐6A/E (Sca‐1) Antibody (108113, BioLegend, USA), PE anti‐mouse CD16/32 Antibody (101307, BioLegend, USA), APC anti‐mouse CD34 Antibody (119309, BioLegend, USA), FITC anti‐mouse CD182 (CXCR2) Antibody (149309, BioLegend, USA), BV421 Mouse Anti‐TCF‐7/TCF‐1 (566692, BD, USA).

### 5.23 Western Blot

Total protein was extracted from mouse tumor tissues (approximately 20 mg) or cultured cells using RIPA lysis buffer (WB3100, NCM Biotech, China) supplemented with a 1% protease and phosphatase inhibitor cocktail (P002, NCM Biotech, China), followed by homogenization (for tissues) or scraping (for cells). Lysates were centrifuged, and the supernatants were collected. Protein concentrations were determined using a BCA assay (G2026, Servicebio, China). Equal amounts of protein were mixed with 5× SDS‑PAGE loading buffer (WB2001, NCM Biotech, China), heated, resolved by SDS‑PAGE on precast gels (ET15412LGel, ACE, China), and transferred onto PVDF membranes (ISEQ00010, Millipore, Germany) using a semi‑dry transfer system (BIO‐RAD, USA). Membranes were blocked with 5% nonfat milk (#9999, CST, USA) in TBST, incubated with primary antibodies overnight at 4°C, followed by HRP‑conjugated secondary antibodies for 1 h at room temperature. Signals were visualized using enhanced chemiluminescence (P10300, NCM Biotech, China) and imaged with a chemiluminescence imaging system (Tanon, China). The primary and secondary antibodies used were as follows: SOCS3 (D6E1T) Rabbit Monoclonal Antibody (52113, CST, USA), Phospho‐Stat3 (Tyr705) (D3A7) Rabbit Monoclonal Antibody (9145, CST, USA), Stat3 (D3Z2G) Rabbit Monoclonal Antibody (12640, CST, USA), Beta Actin Polyclonal antibody (20536‐1‐AP, Proteintech, USA), Goat Anti‐Rabbit IgG (H+L) HRP (S0001, Affinity, China).

### 5.24 CD8+ T Cell Depletion

Starting from day 30 after tumor resection in mice, during contralateral tumor rechallenge, 200 µg of anti‐CD8α neutralizing antibody (Clone: S‐R540) (S0B1141, STARTER, China) was administered via intraperitoneal injection every four days to deplete CD8+T cells, with an equal volume of IgG2a κ (Clone: S‐R483) (S0B0932, STARTER, China) serving as the isotype control.​ CD8+ T cell depletion was verified by flow cytometry analysis, as shown in Figure S1C.

### 5.25 Neutrophil Depletion

Starting two days before tumor resection, mice received intraperitoneal injections of 300 µg anti‐Ly6G neutralizing antibody (Clone: S‐R481) (S0B0981, STARTER, China) every 72 h to deplete neutrophils, while control mice were injected with an equal volume of PBS. Neutrophil depletion was verified by flow cytometry analysis, as shown in Figure 4B.

### 5.26 Clodronate Liposomes Administration

Starting two days before tumor resection, mice received intraperitoneal injections of 200 µL (first injection) or 100 µL (subsequent injections) Clodronate liposomes (C283512, Biotarget, Netherlands) every 72 h to deplete neutrophils, while control mice were injected with an equal volume of PBS.

### 5.27 Paclitaxel Administration

Starting 2 days before tumor resection, mice received intraperitoneal injections of 300 µg paclitaxel (HY‐B0015, MCE, USA) every 72 h for systemic chemotherapy.

### 5.28 In vivo CXCL3 Antagonism

In both neoadjuvant and adjuvant settings, mice were intraperitoneally administered anti‐CXCL3 polyclonal antibody (AF5568, R&D, USA) (2 mg/kg) every 72 h. The dose was selected based on a previous report [85] and confirmed by preliminary experiments showing effective neutrophil reduction in our model.

### 5.29 In Vivo CXCL3 Administration

Recombinant mouse CXCL3 protein (HY‐P7153, MCE, USA) (dissolved in PBS containing 0.1% BSA) was intravenously administered to mice at a dose of 150 µg/kg every 48 h for a total of three injections, while control mice received an equal volume of PBS containing 0.1% BSA. Following each injection, the mice were closely monitored for signs of distress or adverse effects. To assess dose‐dependent effects, recombinant CXCL3 was administered at graded doses (15, 50, 150, 450, and 1350 µg/kg) with the same frequency and duration as described above, and the proportions of total and TGFβ1+ neutrophils in peripheral blood and spleen were evaluated.

### 5.30 In Vivo Monotherapy or Combination Therapy with Anti‐CXCL3 and Anti‐PD‐1 Antibodies

Beginning on day 4 after subcutaneous inoculation or tail vein injection of Py8119 cells, intraperitoneal administration of anti‐CXCL3 polyclonal antibody (2 mg/kg) and anti‐PD‐1 antibody (S0B0594, STARTER, China) (200 µg) as monotherapy or combination therapy was performed every 4 days. For the anti‐CXCL3 antibody, PBS was used as a control, while for the anti‐PD‐1 antibody, mouse IgG1 κ (S0B0788, STARTER, China) served as the isotype control.

### 5.31 SB225002 Administration

For CXCR2 blockade in the Py8119 high burden tumor model in vivo, SB225002 (HY‐16711, MCE, USA) (1 mg/kg) was administered intraperitoneally once daily, together with PBS or anti‑CXCL3 antibody every 72 h. For in vitro neutrophil treatment, SB225002 was used at 50 nM and recombinant mouse CXCL3 protein (HY‑P7153, MCE, USA) at 50 ng/mL for 12 h.

### 5.32 In Vivo Anti‑CXCR6 Antibody Administration

Starting from the day of contralateral tumor rechallenge (day 30 post‑resection), a CXCR6 antibody (BE0463, BioXcell, USA) (100 µg) was administered intraperitoneally every 4 days for a total of four injections.

### 5.33 Flow Cytometric Sorting and Adoptive Transfer of CXCR6+CD8+ T Cells

CXCR6+CD8+ T cells were isolated from mouse splenocytes by flow cytometric staining for CXCR6 and CD8, and sorted using a MoFlo Astrios EQ cell sorter (Beckman Coulter, USA). A total of 2 × 10^5^ sorted cells were adoptively transferred into each recipient mouse via tail vein injection. The antibodies used are listed below: APC CD8a Monoclonal Antibody (17‐0081‐82, eBioscience, Thermo Fisher Scientific, USA), PE anti‐mouse CD186 (CXCR6) Antibody (151103, BioLegend, USA).

### 5.34 TGFβ1 Stimulation of CXCR6+CD8+ TRT Cells In Vitro

CXCR6+CD8+ TRT cells were isolated by flow cytometric sorting and cultured in anti‑CD3ε (100340, BioLegend, USA) (5 µg/mL)‑coated plates with soluble anti‑CD28 (102116, BioLegend, USA) (1 µg/mL). Cells were treated with or without recombinant mouse latent TGFβ1 (UA040386, UA‑Bio, China) at 10 ng/mL (after acid activation). After 48 h, cells were harvested, and TCF7 expression was assessed by flow cytometry.

### 5.35 Lentiviral Transduction

Wild‐type Py8119 cells were transduced with lentivirus carrying the plasmid HBLV‐c‐tfr‐ova‐HIS‐NEO (Hanbio, China) at a multiplicity of infection (MOI) of 0.1. Following transduction, cells were selected with G418 (ST081, Beyotime, China) (400 µg/mL) for two weeks to establish the Py8119‐OVA stable cell line, which was subsequently validated by flow cytometry.

### 5.36 Cxcl3 Knockout in Py8119 Cells

To generate Py8119‐Cxcl3^−/−^ cells, two guide RNAs targeting exon 1 and exon 3 of the mouse Cxcl3 gene (NCBI Gene ID: 330122) were designed using the CRISPR design tool (http://crispr.mit.edu). The guide RNA sequences were as follows: AGCGCGGGAGCTGGTGCGAA GGG and GATAATTAATTAGCCTACTG TGG. Ribonucleoprotein complexes comprising the guide RNAs and Cas9 protein were electroporated into Py8119 cells using the Neon transfection system (Thermo Fisher Scientific, USA) according to the manufacturer's instructions. Single‑cell clones were isolated and expanded, and targeted mutations were verified by PCR using primers flanking the cleavage sites (Forward: 5’‑TCTCTATTGGCCTCTCGCCA‑3’; Reverse: 5’‑ACCACGGACCTGCTTTTTAGA‑3’), with knockout bands identified by PCR and confirmed by Sanger sequencing (GENEWIZ, China). The sequencing results were aligned using Snapgene software to confirm successful knockout. Clone 1C3 was confirmed as a homozygous knockout by PCR and Sanger sequencing, and was selected for maintenance in Ham's F‑12K complete medium under standard culture conditions.

### 5.37 Conditioned Medium Treatment of Neutrophils and CD8+ T Cells

Low and high burden tumor conditioned media (CM‑L and CM‑H) were collected from Py8119 cells cultured under low and high density conditions, respectively, after 4 h of culture in fresh medium. Neutrophils were isolated from mouse bone marrow using MojoSort Mouse Neutrophil Isolation Kit (480057, BioLegend, USA) according to the manufacturer's instructions. Neutrophils were treated with CM‑L or CM‑H for 12 h, followed by flow cytometric analysis of their phenotypes, with or without CXCL3 antibody (PA5‐47756, Thermo Fisher Scientific, USA) (1 µg/mL). Neutrophil conditioned media (N‑CM‑L and N‑CM‑H) were then collected after an additional 4 h culture in fresh medium. OT‑1 CD8+ T cells (purchased from Cyagen) were isolated from mouse spleens using Dynabeads Untouched Mouse CD8 Cells Kit (11417D, Thermo Fisher Scientific, USA), stimulated with SIINFEKL peptide (HY‐P1489, MCE, USA) (1 µg/mL) in the presence of recombinant IL2 (51061‐MNAE, SinoBiological, China) (30 U/mL), and cultured with N‑CM‑L or N‑CM‑H for 48 h. T cell proliferation was assessed by CFSE (HY‐D0938, MCE, USA) dilution, and IFN‑γ production was evaluated by flow cytometry following stimulation with a cell stimulation cocktail containing protein transport inhibitors (C4523, Beyotime, China), with or without TGFβ1 antibody (MAB2402, R&D, USA) (1 µg/mL). To exclude a direct effect of CXCL3 on CD8+ T cells, OT‑1 CD8+ T cells stimulated with SIINFEKL peptide were additionally cultured with or without recombinant mouse CXCL3 protein (50 ng/mL) for 48 h, followed by flow cytometric analysis of their activation phenotypes.

### 5.38 Statistical Analyses

In single‐cell RNA and TCR sequencing analyses, the Student's *t*‐test or Wilcoxon rank sum test was applied for comparison between groups. All statistical analyses related to single‐cell RNA/TCR‐seq and bulk RNA‐seq data were performed using R software and R packages (as detailed above).

For in vivo and in vitro experimental data, continuous variables were compared between groups using the Student's *t*‐test or one‐way ANOVA, while categorical variables were compared using the chi‐square test or Fisher's exact test. For one‑way ANOVA, the Bonferroni post‑hoc test was applied. For survival comparisons, Kaplan–Meier curves and log‐rank tests were used. Unless otherwise specified, all hypothesis tests were two‐tailed. A *p*‐value of < 0.05 was considered statistically significant. The statistical analyses were conducted using GraphPad Prism software (version 10.1.2, USA).

## Author Contributions

**Ji Wang**: methodology, investigation, validation, formal analysis, data curation, writing – original draft, visualization. **Xinyu Tang**: methodology, investigation, validation, formal analysis, writing – review and editing. **Zeju Li**: investigation, visualization, software, writing – review and editing. **Qi Qi**: methodology, investigation, validation, formal analysis, writing – review and editing. **Chang Sun**: methodology, investigation, validation, formal analysis, writing – review and editing. **Wen Sun**: software, investigation, visualization, methodology. **Weiya Zhang**: investigation, visualization. **Yue Yin**: investigation, visualization. **Ya Deng**: investigation, visualization. **Peiwen Ling**: methodology, investigation. **Yifan Xu**: software, visualization. **Guangshun Sun**: visualization, software. **Yi Ren**: methodology, writing – review and editing, conceptualization, investigation, data curation, validation. **Hong Pan**: conceptualization, resources, writing – review and editing, supervision, project administration, funding acquisition. **Shui Wang**: conceptualization, resources, writing – review and editing, supervision, funding acquisition, project administration. **Wenbin Zhou**: conceptualization, resources, writing – review and editing, project administration, supervision, funding acquisition.

## Funding

This work was supported in part by the National Natural Science Foundation of China (82573466, 82573628, and 82573634), the Natural Science Foundation of Jiangsu Province (BK20230017 and BK20251950), and the Postgraduate Research & Practice Innovation Program of Jiangsu Province (KYCX25_2162).

## Conflicts of Interest

The authors declare no conflicts of interest.

## Supporting information




**Supporting File**: advs77976‐sup‐0001‐SuppMat.docx.

## Data Availability

The single‐cell RNA/TCR‐seq and bulk RNA‐seq data generated in this study have been deposited in the Genome Sequence Archive in the National Genomics Data Center (accession number: CRA031254), which are publicly accessible at https://ngdc.cncb.ac.cn/gsa.
