# Supplementary material for: CXCL3 Antagonism Reverses Systemic Antitumor Immune Memory Impairment Associated With High Tumor Burden and Sensitizes Anti‐PD‐1 Therapy
Source: Adv Sci (Weinh). 2026 Sep 27:e77976. Online ahead of print. doi: 10.1002/advs.77976 (PMC13616393; doi:10.1002/advs.77976)
Supplement: Supplementary file 1 [file ADVS-9999-e77976-s001.docx]

Supporting Information

CXCL3 antagonism reverses systemic anti-tumor immune memory impairment associated with high tumor burden and sensitizes anti-PD-1 therapy

Ji Wang, Xinyu Tang, Zeju Li, Qi Qi, Chang Sun, Wen Sun, Weiya Zhang, Yue Yin, Ya Deng, Peiwen Ling, Yifan Xu; Guangshun Sun, Yi Ren*, Hong Pan*, Shui Wang*, Wenbin Zhou*

**Figure S1.** Supplementary data for systemic anti-tumor immune memory phenotypes under low and high tumor burden. **A)** Comparison of exhaustion and memory scores of peripheral blood CD8+ T cells between healthy controls and non-TNBC patients. **B)** Comparison of exhaustion, activation, and progenitor exhaustion scores of peripheral blood CD8+ T cells between pT1 and pT2 patients. **C)** Flow cytometry validation of CD8a depletion efficacy in spleen and peripheral blood mononuclear cells from Py8119 tumor-bearing mice. **D)** Experimental design for pulmonary metastasis formation via tail vein rechallenge following primary tumor resection in low and high burden mice. **E)** Comparison of pulmonary metastatic foci numbers between low and high burden groups in D), with representative HE images. **F)** Tumor growth curves and representative tumor images of rechallenged tumors in low and high burden models established with the same number of Py8119 cells but different growth durations (14 days vs 28 days). ns not significant, *P < 0.05, **P < 0.01, ***P < 0.001, ****P < 0.0001. TNBC, triple-negative breast cancer; HE, hematoxylin-eosin.

**Figure S2.** Indicators related to surgical procedures of tumor resection and postoperative physiological status in mice. **A)** Representative photographs of surgical incisions after tumor resection in mice bearing low or high burden Py8119 tumors, with comparison of incision lengths between the two groups (n = 5 per group). **B)** Representative photographs of tumors with low and high burden in Py8119 and MC38 models. **C)** Comparison of body weight, CRP, white blood cell count, neutrophil percentage, lymphocyte percentage, monocyte percentage, ALT, and AST in mice with low and high tumor burden at day 3 post-resection (n = 5 per group). ns not significant, *P < 0.05, **P < 0.01, ***P < 0.001, ****P < 0.0001. CRP, C-reaction protein; ALT, alanine aminotransferase; AST, aspartate transaminase.

**Figure S3.** Supplementary scRNA/TCR-seq analysis of mouse spleens. **A)** UMAP visualization of coarse cell clustering in single-cell sequencing analysis. **B)** Cell counts and proportions of coarse cell clusters in single-cell sequencing data from low and high burden groups at baseline, day 14 post-resection, and day 30 post-resection. **C)** Dotplot of representative markers for each coarse cell cluster. **D)** Comparison of T cell activation pathway functional scores of overall Cd8+ T cells between low and high burden groups at three time points in B). **E)** Comparison of T cell memory pathway functional scores of overall Cd8+ T cells between low and high burden groups at three time points in B). **F)** Dotplot of representative markers for Cd8+ T cell subsets. **G)** GSEA plot of Cxcr6+Cd8+ T cells in tissue resident pathway. **H)** UMAP distribution of Cxcr6+Cd8+ T cells in tumor reactivity, cytotoxicity, and clonality across three time points in B) in low and high burden groups. **I)** Comparison of Pdcd1, Havcr2, and Lag3 expression in Cxcr6+Cd8+ T cells between low and high burden groups at the three time points. **J)** Distribution of Cd8+ T cell subsets along pseudotime trajectories and their corresponding clonality patterns. **K)** Trends of cytotoxicity scores of Cxcr6+Cd8+ TRT cells along pseudotime trajectories after tumor resection in low and high burden groups. TCR, T cell receptor; UMAP, uniform manifold approximation and projection; GSEA, gene set enrichment analysis.

**Figure S4.** Supplementary data for the critical role of CXCR6+CD8+ TRT cells in the differential systemic immune memory between low and high burden groups. **A)** Flow cytometry analysis comparing the percentage of splenic CD8+ T cells among CD45+ cells between low and high burden groups at baseline, day 14 post-resection, and day 30 post-resection in Py8119 and MC38 models (n = 6 per group). **B-C)** Flow cytometry analysis comparing the percentage of splenic CD44^hi^CD62L^hi^ T cells among CD39+CD8+ cells between low and high burden groups at day 30 post-resection in Py8119 and MC38 model, with representative pseudocolor plots (n = 6 per group). **D-E)** Representative single-channel and corresponding merged images of CD44+CD39+CD8+ T cells at baseline and day 14 post-resection, and CD62L+CD39+CD8+ T cells at day 30 post-resection in the spleens of mice in low and high burden groups in Py8119 and MC38 model at baseline, day 14 post-resection, and day 30 post-resection. White arrows indicate the aforementioned cells. Scale bar: 50 µm. **F)** Representative single-channel and corresponding merged images of CD39+CD8+ T cells in primary and rechallenged Py8119 tumors. Scale bar: 50 µm. **G)** Experimental design, tumor growth curves, and tumor images of rechallenged tumors following resection in the low burden, high burden, and low burden + αCXCR6 groups (n = 6 per group). **H)** Experimental design, tumor growth curves, and tumor images of rechallenged tumors following resection in the low burden, high burden, and high burden + adoptive transfer of CXCR6+CD8+ T cells sorted from low burden donors (n = 6 per group). **I)** Experimental design, tumor growth curves, and tumor images of naïve mice receiving adoptive transfer of CXCR6+CD8+ T cells sorted from low or high burden donors, followed by tumor challenge (n = 6 per group). ns not significant, *P < 0.05, **P < 0.01, ***P < 0.001, ****P < 0.0001. For one‑way ANOVA, Bonferroni post‑hoc test was applied.

**Figure S5.** Comparison of B cell characteristics between low and high burden groups in single-cell analysis. **A)** UMAP visualization of B cells stratified by tumor reactivity (defined primarily by Cd38 and Cr2). **B)** UMAP visualization of Cd38 and Cr2 expression patterns in B cells. **C)** Dotplot comparing expression of Cd38, Cr2, and Cd27 between tumor reactive and non-tumor reactive B cells. **D-E)** Violin plots comparing activation, proliferation, and antigen processing and presentation scores in overall B cells and tumor reactive B cells between low and high burden groups.

**Figure S6.** Comparison of Cd4+T cell characteristics between low and high burden groups in single-cell analysis. **A)** UMAP visualization of Cd4+ T cell subsets and their TCR clonalities (clonal or unique clones). **B)** Dotplot of representative markers for Cd4+ T cell subsets. **C)** Proportions of Cd4+ T cell subsets in low and high burden groups. **D-G)** Violin plots comparing activation, proliferation, and cytokine production scores of overall Cd4+ T cells, Cxcr3+Cd4+ Th1 cells, Maf2+Cd4+Th1 cells, and Isg15+Cd4+ Th1 cells between low and high burden groups.

**Figure S7.** Supplementary analysis for phenotypes of myeloid cells. **A)** Dotplot of representative markers for myeloid cell subsets. **B)** Violin plots comparing immune-suppressive scores of myeloid cell subsets between low and high burden groups at baseline. **C)** GSEA plot of MDSC signature of neutrophils in high burden versus low burden groups at baseline. **D)** Dotplot showing differential expression of Cd274, S100a8, S100a9, Tgfb1, Arg1, and Il1b in neutrophils between low and high burden groups at baseline, day 14 post-resection, and day 30 post-resection. **E-F)** Heatmap of CellChat outgoing and incoming signaling patterns in low and high burden groups for crosstalk among myeloid subsets at baseline, neutrophils at day 14 post-resection, and neutrophils at day 30 post-resection. **G)** Gene expression profiles in CXCL-mediated interactions between neutrophils from low and high burden groups. **H)** Interaction intensity of outgoing and incoming interaction strength between myeloid subsets in E) and F) in low and high burden groups. **I-J)** Representative single-channel and corresponding merged images of immunosuppressive TGFβ1+Ly6G+ neutrophils in the spleen of mice in low and high burden groups in Py8119 and MC38 model at baseline, day 14 post-resection, and day 30 post-resection. White arrows indicate the aforementioned cells. Scale bar: 50 µm. **K)** Expression of Cd274, Tgfb1, Arg1, and Il1b in macrophages, monocytes, cDCs, and pDCs between low and high burden groups at the three time points. **L-M)** mIF showing the spatial localization of macrophages, neutrophils, and CD8+ T cells, along with TGFβ1 expression, in primary tumors and spleens. **N)** Comparison of TGFβ1 producing‑related pathway scores in neutrophils, macrophages, and Treg cells at baseline in mouse scRNA‑seq data. **O)** Heatmap showing the expression of Tcf7, Smad3, Eomes, and Tbx21 in Cxcr6+Cd8+ TRT cells between low and high burden groups at the three time points based on scRNA‑seq data, and comparison of TCF7+ cell frequencies in sorted CXCR6+CD8+ TRT cells following in vitro treatment with or without TGFβ1. **P)** Representative single-channel and corresponding merged images of TGFβ1+ neutrophils in primary and rechallenged Py8119 tumors. Scale bar: 50 µm. **Q)** Comparison of CXCR6 and ENTPD1 expression in CD8+ T cells and TGFB1 expression in neutrophils between pT1 and pT2 patients based on peripheral blood scRNA‑seq data from breast cancer patients. **R)** Experimental design of PBS, neutrophil depletion, and macrophage depletion around the time of tumor resection. **S)** Comparison of splenic CD39+CD8+ T cell frequencies and Ki67+ proportions among the three groups in R) at day 14 post‑resection, and CD39+CD8+ T cell frequencies and CD44^hi^CD62L^hi^ proportions at day 30 post‑resection. **T)** Expression of H2-Ab1, H2-Aa, H2-Eb1, Cd80, Cd86, and Cd83 in cDCs and pDCs between low and high burden groups at the three time points. ns not significant, *P < 0.05, **P < 0.01, ***P < 0.001, ****P < 0.0001. For one‑way ANOVA, Bonferroni post‑hoc test was applied. GSEA, gene set enrichment analysis; MDSC, myeloid-derived suppressor cell.

**Figure S8.** Peripheral blood validation of splenic immunophenotypes in low and high burden groups. **A)** Experimental design of flow cytometry analysis for immune cells in peripheral blood of high and low burden groups at baseline, day 14 post-resection, and day 30 post-resection. **B)** Flow cytometry analysis comparing the percentage of CD8+ T cells among CD45+ cells in peripheral blood between low and high burden groups at three time points in A) (n = 6 per group). **C)** Flow cytometry analysis comparing the percentage of CD39+CD8+T cells among CD45+ cells in peripheral blood between low and high burden groups at three time points in A) (n = 6 per group). **D)** Flow cytometry analysis comparing the percentages of Ki67+ and IFNγ+ cells among CD39+CD8+T cells in peripheral blood between low and high burden groups at baseline and day 14 post-resection, as well as the percentages of CD62L^hi^ and IL7Rα^hi^ at day 30 post-resection (n = 6 per group). **E)** Flow cytometry analysis comparing the percentage of CD44^hi^CD62L^hi^CD8+T cells among CD39+CD8+cells in peripheral blood between low and high burden groups at day 30 post-resection, with representative pseudocolor plots (n = 6 per group). **F)** Flow cytometry analysis comparing the percentage of neutrophils among CD45+ cells in peripheral blood between low and high burden groups at three time points in A) (n = 6 per group). **G)** Flow cytometry analysis comparing the percentage of TGFβ1+ neutrophils among neutrophils in peripheral blood between low and high burdens groups at three time points in A) (n = 6 per group). ns not significant, *P < 0.05, **P < 0.01, ***P < 0.001, ****P < 0.0001.

**Figure S9.** Supplementary analysis of the role of CXCL3 or CXCL3 antagonism in systemic anti-tumor immune status. **A-B)** Comparison of the proportions of TGFβ1+CXCR2+ neutrophils and CD34+CD16/32+ granulocyte‑monocyte progenitors in the bone marrow between low and high burden groups. **C)** Enrichment changes of NF‑κB, JAK‑STAT, and HIF‑1 pathways in high versus low burden groups in both in vitro and in vivo settings based on bulk RNA‑seq data. **D)** Western blot validation of p‑STAT3, STAT3, and SOCS3 expression in high versus low burden groups in both in vitro and in vivo settings. **E)** Heatmap showing the expression differences of Cxcl9, Cxcl10, and Ccl5 between low and high burden groups in both in vitro and in vivo settings based on bulk RNA‑seq data. **F)** Comparison of Cxcl9, Cxcl10, and Ccl5 expression in cDCs, pDCs, monocytes, macrophages, and neutrophils between low and high burden groups at the three time points based on scRNA‑seq data. **G)** Comparison of Cxcr3 and Ccr5 expression in overall CD8+ T cells and Cxcr6+CD8+ T cells between low and high burden groups at the three time points based on scRNA‑seq data. **H)** Experimental design of recombinant CXCL3 protein supplementation in tumor-free mice. **I-J)** Comparison of proportions neutrophils and TGFβ1+ neutrophils in the spleen and peripheral blood of tumor-free mice following PBS or CXCL3 administration (n = 6 per group). **K)** Comparison of TGFβ1+ neutrophils in the spleen via mIF in tumor-free mice following PBS or CXCL3 administration (n = 6 per group). Scale bar: 50 µm. **L-M)** Comparison of proportions of neutrophils and TGFβ1+ neutrophils in the spleen and peripheral blood of tumor‑free mice following tail vein injection of PBS or graded doses of recombinant CXCL3 protein (n = 6 per group). **N)** Experimental design and tumor growth curves with tumor images of high burden Py8119 tumors treated with PBS or αCXCL3 in the presence of SB225002 (n = 8 per group). **M)** Comparison of proportions of CD39+CD8+ T cells and TGFβ1+ neutrophils in the spleens and peripheral blood between Py8119‑Cxcl3^⁻/⁻^ and wild‑type groups (n = 6 per group). **O)** Comparison of TGFβ1+ neutrophil proportions following in vitro treatment with recombinant CXCL3, with or without SB225002. **P)** Comparison of CD39+CD8+ T cell frequencies and Ki67+ proportions among CD39+CD8+ T cells between Py8119‑Cxcl3^⁻/⁻^ and wild‑type groups at day 14 post‑resection, and CD39+CD8+ T cell frequencies and CD44^hi^CD62L^hi^ proportions among CD39+CD8+ T cells at day 30 post‑resection in spleens and peripheral blood. **Q)** Tumor growth curves and tumor images on day 20 after re‑challenge in low and high burden Py8119‑Cxcl3^⁻/⁻^ models following resection (n = 6 per group). **R-S)** Bar graph and representative pseudocolor plots comparing the proportions of OVA-specific (tetramer+) CD8+ T cells in spleens and peripheral blood across different groups at day 14 post-resection, following systemic administration of PBS or CXCL3 antagonism under adjuvant/neoadjuvant settings (n = 6 per group). **T-U)** Bar graph comparing the proportions of Ki67+, PD-1+, and Tim3+ cells among OVA-specific (tetramer+) CD8+ T cells in spleens and peripheral blood across different groups at day 14 post-resection, following systemic administration of PBS or CXCL3 antagonism under adjuvant/neoadjuvant settings (n = 6 per group). **V)** Analysis of CXCL3 expression differences in breast cancer patients across different T stages, along with subgroup analyses in Luminal and Non-luminal subtypes, using the METABRIC database. ns not significant, *P < 0.05, **P < 0.01, ***P < 0.001, ****P < 0.0001. For one‑way ANOVA, Bonferroni post‑hoc test was applied. OVA, ovalbumin.

**Figure S10.** Main gating strategies for flow cytometry. **A)** Gating strategy for CD39+CD8+ T cells and their activation/proliferation markers IFNγ and Ki67. **B)** Gating strategy for CD39+CD8+ T cells and their memory markers CD44, CD62L, and IL7Rα. **C)** Gating strategy for neutrophils, TGFβ1+ neutrophils, and TGFβ1+CXCR2+ neutrophils. **D)** Gating strategy for tetramer+CD8+ T cells (OVA-specific) and their markers (Ki67, PD-1, Tim3, CD44^hi^CD62L^hi^, IL7Rα). **E)** Gating strategy for granulocyte‑monocyte progenitors in mouse bone marrow cells. **F)** Gating strategy for TCF7+ cells in sorted CXCR6+CD8+ T cells. **G)** Gating strategy for Ki67+ cells in sorted OT1 CD8+ T cells.
